# Endothelial Dickkopf-1 Promotes Smooth Muscle Cell-derived Foam Cell Formation via USP53-mediated Deubiquitination of SR-A During Atherosclerosis

**DOI:** 10.7150/ijbs.91957

**Published:** 2024-05-19

**Authors:** Xiaolin Liu, Tengfei Zheng, Yu Zhang, Yachao Zhao, Fengming Liu, Shen Dai, Meng Zhang, Wencheng Zhang, Cheng Zhang, Mei Zhang, Xiao Li

**Affiliations:** 1 National Key Laboratory for Innovation and Transformation of Luobing Theory; The Key Laboratory of Cardiovascular Remodeling and Function Research, Chinese Ministry of Education, Chinese National Health Commission and Chinese Academy of Medical Sciences; Department of Cardiology, Qilu Hospital of Shandong University, Jinan, China.; 2 Department of Immunology, School of Basic Medical Sciences, Shandong University, Jinan, China.; 3 Department of Physiology & Pathophysiology, School of Basic Medical Sciences, Shandong University, Jinan, China.

**Keywords:** shear stress, atherosclerosis, Dickkopf-1, cellular communication, foam cell

## Abstract

**Background:** Shear stress-induced Dickkopf-1 (DKK1) secretion by endothelial cells (ECs) promotes EC dysfunction and accelerates atherosclerosis (AS). However, the paracrine role of endothelial DKK1 in modulating adjacent smooth muscle cells (SMCs) in atherosclerosis remains unclear. This study investigated the role of EC-secreted DKK1 in SMC-derived foam cell formation under shear stress, *in vitro* and *in vivo*.

**Methods:** Parallel-plate co-culture flow system was used to explore the cellular communication between ECs and SMCs under shear stress *in vitro*. Endothelium-specific knockout of DKK1 (DKK1^ECKO^/APOE^-/-^) and endothelium-specific overexpression of DKK1 (DKK1^ECTg^) mice were constructed to investigate the role of endothelial DKK1 in atherosclerosis and SMC-derived foam cell formation *in vivo*. RNA sequencing (RNA-seq) was used to identify the downstream targets of DKK1. Reverse transcription quantitative polymerase chain reaction (RT-qPCR), western blot, coimmunoprecipitation (Co-IP) assays and chromatin immunoprecipitation (ChIP) experiments were conducted to explore the underlying regulatory mechanisms.

**Results:** DKK1 is transcriptionally upregulated in ECs under conditions of low shear stress, but not in co-cultured SMCs. However, DKK1 protein in co-cultured SMCs is increased via uptake of low shear stress-induced endothelial DKK1, thereby promoting lipid uptake and foam cell formation in co-cultured SMCs via the post-translational upregulation of scavenger receptor-A (SR-A) verified in parallel-plate co-culture flow system, DKK1^ECKO^ and DKK1^ECTg^ mice. RNA sequencing revealed that DKK1-induced SR-A upregulation in SMCs is dependent on Ubiquitin-specific Protease 53 (USP53), which bound to SR-A via its USP domain and cysteine at position 41, exerting deubiquitination to maintain the stability of the SR-A protein by removing the K48 ubiquitin chain and preventing proteasomal pathway degradation, thereby mediating the effect of DKK1 on lipid uptake in SMCs. Moreover, DKK1 regulates the transcription of USP53 by facilitating the binding of transcription factor CREB to the USP53 promoter. SMC-specific overexpression of USP53 via adeno-associated virus serotype 2 vectors in DKK1^ECKO^/APOE^-/-^ mice reversed the alleviation of atherosclerotic plaque burden, SR-A expression and lipid accumulation in SMCs within plaques resulting from DKK1 deficiency.

**Conclusions:** Our findings demonstrate that, endothelial DKK1, induced by pathological low shear stress, acts as an intercellular mediator, promoted the foam cell formation of SMCs. These results suggest that targeted intervention with endothelial DKK1 may confer beneficial effects on atherosclerosis.

## Introduction

Atherosclerosis (AS) is a chronic progressive inflammatory disease characterized by excessive lipid accumulation and plaque formation 1. Atherosclerotic plaques develop primarily at the curvatures and bifurcations of arteries, where blood flow becomes turbulent, leading to atheroprone oscillatory shear stress 2. The distribution of AS plaques is related to local oscillations in shear stress 3, 4. ECs and SMCs are the main cellular components of the vascular wall. ECs comprise the innermost layer of blood vessels and are directly exposed to mechanical forces, primarily shear stress. In addition, ECs convert mechanical stimuli into intracellular signals that communicate with adjacent SMCs. The interaction between ECs and SMCs is essential for maintaining the structure and function of blood vessels, and for vascular remodeling 5, 6. ECs secrete inflammatory factors and vasoactive substances in response to shear stress and regulate SMC function through paracrine mechanisms 6. However, further studies are required to elucidate the role of intercellular interactions between ECs and SMCs in pathological shear stress-induced AS.

AS is characterized by the continuous aggregation and accumulation of plasma lipoproteins in the intima of blood vessels, which are oxidized, modified, and phagocytosed by macrophages or SMCs to form cholesterol-rich foam cells 7-9. Historically, research on foam cells has primarily focused on macrophages because of their central role in driving plaque development 10. Following recent developments in lineage-tracing technology and single-cell genomics, pioneering studies have confirmed that SMCs also contribute significantly to foam cell populations within plaques 11. SMC-derived foam cells constitute at least 50% of human coronary atherosclerotic plaques 12. In a murine model of atherosclerotic plaques, SMC-derived foam cells were found to account for 70% of the total foam cell population during the early and middle stage of AS in APOE^-/-^ mice 13. Furthermore, acquisition of the macrophage marker CD68 by SMCs within plaques led to the identification of foam cells derived from SMCs exhibiting a macrophage-like phenotype 12. Thus, SMCs may have a greater role in the formation of foam cell populations than previously recognized. Additionally, injured ECs—the initial trigger for atherosclerotic plaque development—release various cytokines and secretory proteins that initiate SMC-derived foam cell formation. Dysfunctional ECs and inflammatory cells secrete chemokines and growth factors to promote the proliferation and migration of SMCs from the media to the intima, where they take up lipoproteins and transform into foam cells. Recent studies have reported that ECs can regulate cholesterol metabolism or reduce cholesterol efflux in co-cultured SMCs in a paracrine manner 14, 15. However, as the primary risk factor determining the distribution of atherosclerotic plaques, the role of pathological shear stress-induced ECs dysfunction in regulating lipid deposition in adjacent SMCs remains unknown.

Dickkopf-1 (DKK1), the most widely studied glycoprotein in the Dickkopf family, is mainly secreted by ECs and platelets, and is a hemodynamically sensitive protein. DKK1 inhibits the classical Wnt pathway and binds to CKAP4 to activate the PI3K/Akt pathway, thereby exerting both autocrine and paracrine effects 16, 17. Several studies have shown that DKK1 is abnormally expressed in a variety of tumors, where it is associated with cell proliferation and apoptosis. The potential for DKK1-neutralizing antibodies as antitumor drugs has been evaluated in clinical trials 18. In recent years, attention has focused on the pivotal roles of DKK1 in the pathogenesis of cardiovascular diseases 19. Notably, studies have shown that DKK1 plasma levels are strongly correlated with AS 20. DKK1 is involved in pathological shear stress-induced ECs dysfunction 21 and plays a crucial role in regulating the proliferation and migration of SMCs in response to mechanical stretch 22. Previously, we found that DKK1 deficiency in APOE^-/-^ mice significantly reduced the lipid content within plaques 23, while there was no inhibitory effect on intracellular lipid accumulation in macrophages 24. This suggested that the role of DKK1 in the development of AS and in plaque lipid deposition is independent of macrophage-derived foam cell formation. Therefore, we propose that DKK1 regulates foam cell formation during atherogenesis via an alternative pathway, possibly involving SMC-derived foam cells. This study is the first to investigate the paracrine effect of EC-induced DKK1 on foam cell formation in SMCs under conditions of pathological shear stress.

This study was designed to evaluate the effects of EC-SMC interactions under shear stress on SMC-derived foam cell formation and AS, and to elucidate the role of DKK1 in this process and its underlying mechanism of action.

## Materials and Methods

Detailed methods are provided in Supplementary Material: Supplementary Methods.

### Human primary cells Culture and Treatment

Human Aortic Endothelial Cells (HAECs), hereinafter referred to as ECs, were obtained from ScienCell Research Laboratories (FC-6100, CA, USA) and cultured in Endothelial Cell Medium (ECM, 1001, ScienCell, USA) at 37°C in 5% CO_2_ incubator. Cells in the 2 to 6 passages were used for experiments and transfected with DKK1-siRNA (Table S3). Human aortic smooth muscle cells (HASMCs), hereinafter referred to as SMCs, were obtained from ScienCell Research Laboratories (FC-0015, CA, USA) and cultured in Smooth Muscle Cell Medium (SMCM, 1152, ScienCell, USA) at 37°C in 5% CO_2_ incubator. Cells in the 2 to 6 passages were used for experiments and transfected with the corresponding siRNA (Table S3) constructed by genepharma Co., LTD (Shanghai, China) at an appropriate OD level or adenoviruses at an appropriate multiplicity of infection (MOI). SMCs were treated with 100 μg/ml oxidized low-density lipoprotein (oxLDL, YB-002, Yiyuan Biotech, Guangdong, China), low density lipoprotein (LDL) or acetylated low-density lipoprotein (acLDL) diluted with free-FBS SMCM for 24 h to induce SMC-derived foam cell formation. Human Embryonic Kidney 293T (HEK293T) Cells were cultured in DMEM (SH30022.01B, Hyclone, USA) with 10% FBS (10099141, Gibco, Grand Island, NY, USA) and 1% penicillin-streptomycin (P1400, Solarbio) and transfected with the corresponding plasmids for 48 h at an appropriate quality.

### Human studies

Human carotid plaque biopsies, used for histological analyses, were collected from patients undergoing carotid endarterectomy at the Department of Neurosurgery at the Qilu Hospital of Shandong University. Informed consent was obtained from all participating patients. All procedures were approved by the Medical Ethics Committee of Shandong University, Jinan, China (Approval ID: KYLL-2020-183) and performed in compliance with the principles of the Declaration of Helsinki. Carotid artery tissues were fixed overnight in 4% paraformaldehyde (PFA), embedded in paraffin, sectioned, and immunochemically stained after antigen retrieval to analyze antigen expression in human atherosclerotic plaques.

### Generation of endothelial-specific DKK1 knockout in APOE^-/-^ mice

Endothelial-specific DKK1 knockout mice (C57BL/6J background) were generated by crossing DKK1^fl/fl^ mice (Viewsolid Biotech Co. Ltd, Beijing, China) with TEK^CreERT2^ (Jackson Laboratory) transgenic mice expressing Cre recombinase under control of the TEK promoter to obtain DKK1^fl/fl^/TEK^CreERT2^ mice. These mice were then intercrossed with APOE^-/-^ mice (Viewsolid Biotech Co. Ltd.) to obtain DKK1^fl/fl^/TEK^CreERT2^/APOE^-/-^ double-knockout mice. Endothelial-specific DKK1 knockout (DKK1^ECKO^) mice were induced by injecting 4-week-old DKK1^fl/fl^/TEK^CreERT2^ mice with tamoxifen (100 mg/kg per day, T-5648, Sigma-Aldrich, St. Louis, MO, USA) every other day for a total of five times. A genotype identification kit was purchased from Bimake Technology (B40013). Genomic DNA was extracted from tail biopsies obtained from 2-week-old mice and the genotype was identified using the following primers: m-APOE F1: 5'-TCTCGGCTCTGAACTACATAGG-3' and m-APOE R1: 3'-TTCGAAGCCAGCTTGAGTTAC-5'; m-DKK1-loxP-tF1: 5'-TATGAGGGCGGGAACAAGTACCAGA-3' and m-DKK1-loxP-tR2: 3'-TGTGGGGGCAGTACTTCTTTGAAGG-5'; Cre F: 5'-CAGCATTGCTGTCACTTGGTC-3' and Cre R: 3'-ATTTGCCTGCATTACCGGTCG-5'. The mice used in this study were maintained under specific pathogen-free (SPF) conditions and fed rodent chow.

### Generation of endothelial-specific DKK1 overexpression mice

The CAG-LSL-DKK1-Wpre-Pa expression frame was inserted into the Rosa26 gene site via homologous recombination using CRISPR/Cas9 technology. Briefly, the Cas9 mRNA and gRNA were obtained via *in vitro* transcription. The donor vector was constructed through in-fusion cloning; it contained 3.3 Kb 5′ homologous arms CAG-LSL-DKK1-wpre-Pa and 3.3 Kb 3′ homologous arms to donor. The Cas9 mRNA gRNA vector was microinjected into the fertilized eggs of C57BL/6J mice. F0 generation mice were confirmed by PCR amplification and sequencing, and then mated with C57BL/6J mice to obtain five positive F1 generation mice. Male transgenic (DKK1^ECTg^) and wild type (WT) mice were bred by Viewsolid Biotech Co. Ltd. AS was induced via tail vein injection of AAV8 encoding the Asp374-to-Tyr mutant version of PCSK9 (PCSK9DY; Weizhen Biotechnology, Shanghai, China, Co., Ltd.), after which mice were fed a HFD diet.

### AAV2-SM22α-USP53-overexpressing mice

Mouse USP53 and corresponding control genes were amplified from cDNA and constructed plasmids were packaged into AAV2 vectors carrying the smooth muscle 22 α (SM22α) promoter to drive the expression of control (AAV2-SM22α-Control) and USP53 (AAV2-SM22α-USP53, Gene ID:99526) genes (provided by Weizhen Biotechnology, Shanghai, China, Co., Ltd). Viral solution (7.83 × 10^13^ vg/mL, 10^12^ vg per mouse diluted in sterile saline) was slowly injected into the tail vein of conscious immobilized mice. Mice were subjected to partial ligation of the left carotid artery at least 4 weeks after the AAV2 injection. All surgery and subsequent analyses were performed in a blinded manner.

### Statistical analysis

All experiments were repeated at least three times. The number of biological repeats for each experimental group is listed in the respective figure legend. Data are presented as the Mean ± Standard Deviation (SD). Two-tailed unpaired Student's *t*-test was used to evaluate differences between two groups. One-way ANOVA followed by Tukey's multiple comparison test was used for multiple group comparisons. Statistical analyses were performed using GraphPad Prism 8.3.0 software. Statistical significance was defined as *p* < 0.05.

## Results

### Disturbed flow stimulates DKK1 expression in vascular cells

**Figure 1:**
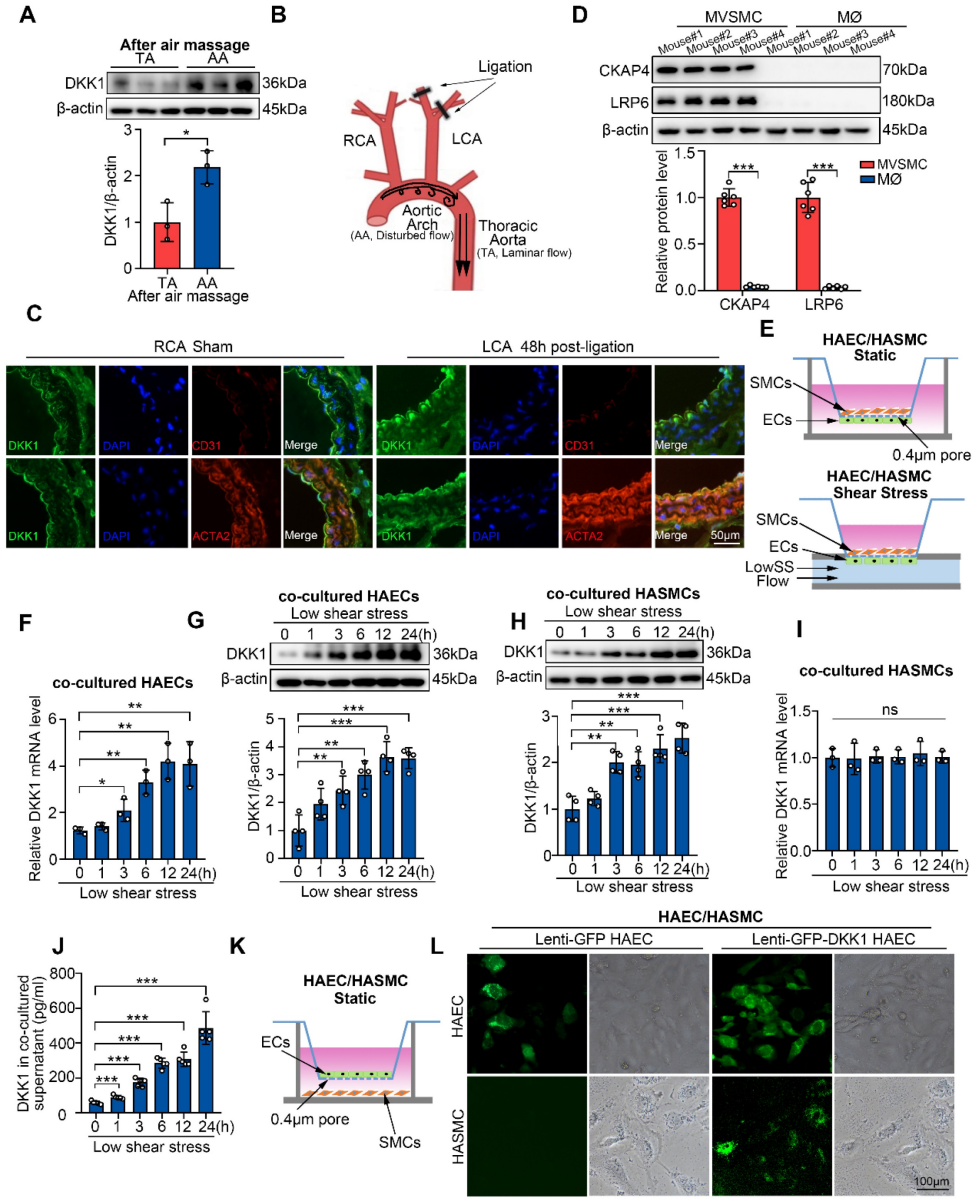
** LowSS upregulated endothelial DKK1 expression *in vivo* and *in vitro*, which could be taken up by smooth muscle cells (SMCs) in a co-culture model. (A)** Western blot and quantification of DKK1 in the aortic arch (AA) and descending thoracic aorta (TA) of C57BL/6J mice after denuding endothelium (n = 3). **(B)** Schematic diagram of the partial carotid ligation model. **(C)** Double immunofluorescence staining for DKK1 (green) and ACTA2 (red) or CD31 (red) in the LCA 48 h following partial ligation in C57BL/6J mice. The un-ligated RCA was used as a sham group. **(D)** Western blot and quantification of CKAP4 and LRP6 in mouse vascular smooth muscle cells (MVSMCs) and macrophages (MØ) (n = 6). **(E)** Schematic diagram of the endothelial cell (EC)/smooth muscle cell (SMC) co-culture and flow system. **(F, G)** mRNA (F, n = 3) and protein (G, n = 4) level of DKK1 in ECs co-cultured under low shear stress (LowSS) for the indicated duration. **(H, I)** DKK1 protein (H, n = 4) and mRNA (I, n = 3) level in SMCs co-cultured under LowSS for the indicated duration.** (J)** Protein expression of DKK1 in the supernatant of co-cultured ECs and SMCs under LowSS for the indicated duration (n = 5). **(K, L)** SMCs were co-cultured with green fluorescent protein (GFP)-labeled-DKK1 transfected human aortic endothelial cells (HAECs) or control and GFP (green) expression in the basement cells was determined. ^*^p < 0.05, ^**^p < 0.01, ^***^p < 0.001, ns not significant. Data are shown as the Mean ± Standard Deviation (SD). Statistical analysis was performed with Two-tailed unpaired Student's *t*-test or One-way ANOVA followed by Tukey's multiple comparison test.

ECs are the main cell type responsible for pathological shear stress-stimulated DKK1 secretion in vascular walls 21, 23. To investigate DKK1 levels in other cell types of the vascular wall under conditions of disturbed flow, the endothelium of vascular walls was denuded to eliminate potential interference from ECs 25. Next, we examined DKK1 levels in the descending thoracic aorta (TA), where physiological shear stress is present, and in the aortic arch (AA), which represents pathological low shear stress, via western blot analysis. Our findings revealed a significant increase in DKK1 expression in EC-denuded AA compared to EC-denuded TA (Figure 1A, S1A). To verify the effect of disturbed flow on DKK1 levels in various cell types of the vascular wall *in vivo*, a mouse model of partial carotid ligation was established to simulate pathological shear stress (Figure 1B). DKK1 expression in the vascular walls was determined by immunofluorescence staining and Western blot 48 h following ligation (Figure 1C, S1B). The expression of DKK1 was significantly higher in the ligated left common carotid artery (LCA) compared with that in the un-ligated right common carotid artery (RCA sham) (Figure S1B). Moreover, immunofluorescence staining revealed that DKK1 expression was increased in both the EC layer marked by CD31 and the adjacent SMC layer marked by ACTA2 of the ligated LCA compared to the un-ligated RCA (Figure 1C). Previous studies in our group have demonstrated that pathological shear stress does not influence the expression of DKK1 in vascular SMCs 21, 23, in combination with the above data, we hypothesized that the increased DKK1 expression in SMCs adjacent to ECs, might be due to EC-secreted DKK1 under conditions of pathological shear stress.

### SMCs take up low shear stress-induced endothelial DKK1 in a co-cultured system

Since both SMCs and macrophages can interact with ECs, and are cellular sources of plaque foam cell populations, we compared the expression of DKK1 receptors, CKAP4 and LRP6, on the surfaces of primary mouse vascular smooth muscle cells (MVSMCs) and macrophages (MØ) through Western blot. MVSMCs expressed high levels of CKAP4 and LRP6, whereas macrophages expressed very low levels of these two DKK1 receptors (Figure 1D). Thus, SMCs may be the primary functional targets of EC-secreted DKK1. Next, we explored DKK1-mediated interactions between ECs and SMCs under shear stress *in vitro*. A parallel-plate co-culture flow system (Figure 1E) was used to explore DKK1 levels in co-cultured ECs and SMCs under shear stress. In a time-course experiment, pathological low shear stress (LowSS, 4 dyne/cm^2^) upregulated DKK1 in ECs from 3 h at both the mRNA and protein levels (Figure 1F, 1G). However, only protein level of DKK1 was significantly elevated from 3 h in co-cultured SMCs (Figure 1H), whereas the mRNA level of DKK1 was not altered (Figure 1I). Furthermore, secretion of DKK1 into the supernatant of the parallel-plate co-culture flow system was increased under the LowSS treatment (Figure 1J). A similar phenomenon was observed in the stress-force-dependent experiment, in which ECs were exposed to static conditions, physiological normal shear stress NSS (12 dyne/cm^2^) or LowSS (4 dyne/cm^2^) for 24 h (Figure S1C-F). Increased secretion of DKK1 into the supernatant was observed in the LowSS-treated group (Figure S1G). These results indicated that LowSS has a direct effect on the expression of DKK1 in ECs rather than in SMCs, whereas increased DKK1 protein expressed in SMCs is secondary to the elevated transcription of DKK1 in ECs rather than in the SMCs themselves. To determine whether SMCs have the ability to take up EC-secreted DKK1, a co-culture system was performed by placing GFP-labeled-DKK1-transfected ECs in the upper chamber and SMCs in the lower chamber (Figure 1K). As shown in Figure 1L, a large amount of GFP-labeled DKK1 was observed in SMCs after 12 h of co-culture. Above all, these findings indicate that SMCs can take up ECs-secreted DKK1 under pathological shear stress conditions, which simulates disturbed flow *in vivo*.

### LowSS-induced endothelial DKK1 promotes lipid uptake by co-cultured SMCs via the upregulation of SR-A

**Figure 2:**
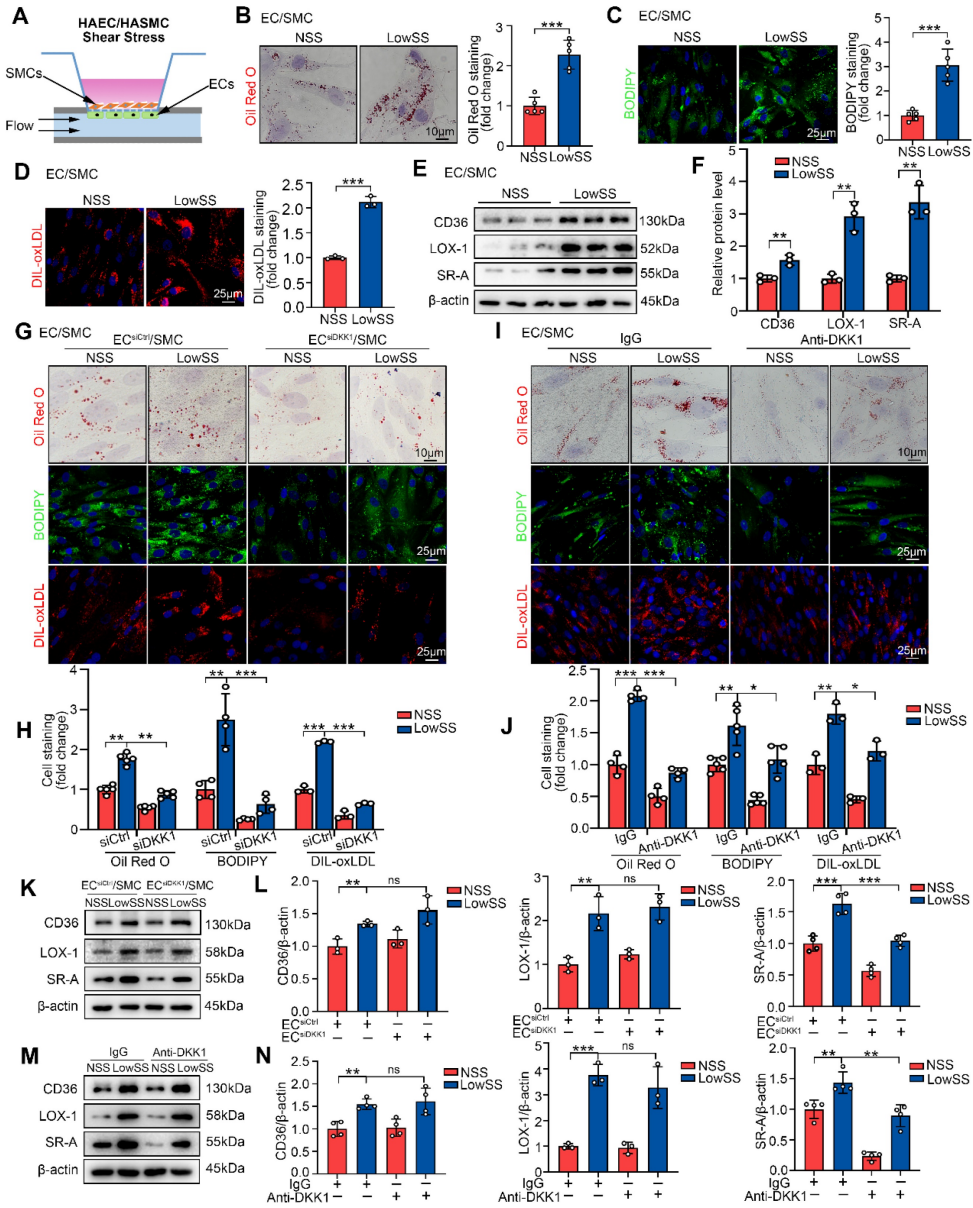
** LowSS induced-endothelial DKK1 promoted foam cell formation and SR-A expression in co-cultured SMCs. (A)** Schematic diagram showing the EC/SMC co-culture flow system.** (B-D)** Representative images and quantification of Oil Red O (B, n = 5), BODIPY493/503 (C, n = 5), and DIL-oxLDL (D, n = 3) staining of SMCs co-cultured with ECs under normal shear stress (NSS) or LowSS for 24 h. **(E, F)** Western blot and quantification of CD36, LOX-1, and SR-A in co-cultured SMCs under shear stress (n = 3). **(G, H)** Oil Red O (upper panel, n = 5), BODIPY493/503 (middle panel, n = 4), DIL-oxLDL (lower panel, n = 3) staining (G) and quantification (H) of SMCs co-cultured with ECs transfected with Control-siRNA (siCtrl) or DKK1 interference siRNA (siDKK1) under shear stress conditions. **(I, J)** Oil Red O (upper panel, n = 4), BODIPY493/503 (middle panel, n = 5), and DIL-oxLDL (lower panel, n = 3) staining (I) and quantification (J) of co-cultured SMCs incubated with a DKK1-neutralizing antibody (10 μg/mL) or control IgG under shear stress conditions. **(K, L)** Western blot (K) and quantification (L) of CD36 (n = 3), LOX-1 (n = 3), and SR-A (n = 4) in SMCs co-cultured with ECs transfected with siCtrl or siDKK1 under shear stress conditions. **(M, N)** Western blot (M) and quantification (N) of CD36 (n = 4), LOX-1 (n = 3), and SR-A (n = 4) in co-cultured SMCs incubated with DKK1-neutralizing antibody or control IgG under shear stress conditions. ^*^p < 0.05, ^**^p < 0.01, ^***^p < 0.001, ns not significant. Data are shown as the Mean ± SD. Statistical analysis was performed with Two-tailed unpaired Student's *t*-test or One-way ANOVA followed by Tukey's multiple comparison test.

To examine the effect of the EC-SMC interaction on foam cell formation of SMCs under shear stress conditions, a parallel-plate co-culture flow system was utilized (Figure 2A, indicated by EC/SMC). First, we established a SMC-derived foam cell model *in vitro*. Consistent with a previous report 26, our findings demonstrated that oxLDL, rather than LDL or acLDL, had a higher propensity for uptake by SMCs and subsequently induced the foam cell phenotype (Figure S2A). Following treatment with oxLDL, co-cultured SMCs in the LowSS-stimulated ECs group exhibited a significant increase in lipid deposition, as evidenced by cellular Oil Red O and BODIPY493/503 staining, and enhanced lipid uptake, as indicated by DIL-oxLDL staining, compared to the NSS-stimulated ECs group (Figure 2B-D). Western blot results showed that under oxLDL conditions, LowSS significantly promoted the expression of CD36, LOX-1, and SR-A in co-cultured SMCs (Figure 2E, 2F), which are transmembrane proteins located on the cell surface that facilitate the recognition and phagocytosis of oxLDL 27. These results indicate that pathological shear stress promotes the formation of SMCs foam cells co-cultured with ECs.

We hypothesized that LowSS-induced foam cell formation of SMCs was mediated by ECs-secreted DKK1. To test this hypothesis, DKK1-siRNA was transfected into ECs (Figure S2B, S2C) and then co-cultured with SMCs under shear stress application. Next, co-cultured SMCs from each group were treated with oxLDL for 24 h or DIL-oxLDL for 4 h. We found that lipid deposition and lipid uptake in co-cultured SMCs were profoundly inhibited by DKK1 knockdown in ECs under LowSS stimulation (Figure 2G, 2H). Western blot analysis showed that knockdown of DKK1 in ECs inhibited LowSS-upregulated SR-A expression in co-cultured SMCs but had no influence on CD36 or LOX-1 expression (Figure 2K, 2L). Furthermore, we used a neutralizing antibody to block DKK1 in the supernatant. The results showed that LowSS-induced lipid accumulation, lipid uptake and SR-A expression in co-cultured SMCs were reversed by the DKK1-neutralizing antibody (Figure 2I, 2J and Figure 2M, 2N). These data indicated that pathological shear stress-induced endothelial DKK1 mediated the foam cell formation of co-cultured SMCs. To further confirm whether DKK1 served as a paracrine mediator secreted by ECs, we incubated SMCs with DKK1 overexpressed-EC medium with or without DKK1 neutralizing antibody. The results showed that DKK1 overexpressed-EC medium induced lipid deposition, DIL-oxLDL uptake, and SR-A expression in SMCs, which were reversed by the DKK1-neutralizing antibody (Figure S3A-C). These data further indicated that endothelial secretion of DKK1 mediated the foam cell formation of SMCs.

Considering the intricate composition of plaque, during which process ECs, SMCs, macrophages and platelets may communicate with each other, we also investigated the effect of endothelial DKK1 on macrophages as well as the influence of platelet-derived DKK1 on SMCs. As shown in our result, LowSS-induced lipid accumulation and lipid uptake in co-cultured macrophages were not affected by knockdown of DKK1 in ECs (Figure S3D). Therefore, these findings suggest that ECs-derived DKK1 has no effect on macrophage foam cell formation. As another major source of DKK1, platelets-SMCs interaction on SMC-derived foam cell formation remained unclear. We found that although DKK1 derived from activated platelets through SFLLRN could enhance lipid accumulation in SMCs (Figure S3E), their impact on SMCs was observed only upon entry into atherosclerotic plaques.

Collectively, these findings suggest that LowSS triggers ECs to secrete DKK1, which promotes oxLDL-induced foam cell formation by upregulating SR-A expression in co-cultured SMCs.

### DKK1 upregulates of SR-A to promote foam cell formation of SMCs

**Figure 3:**
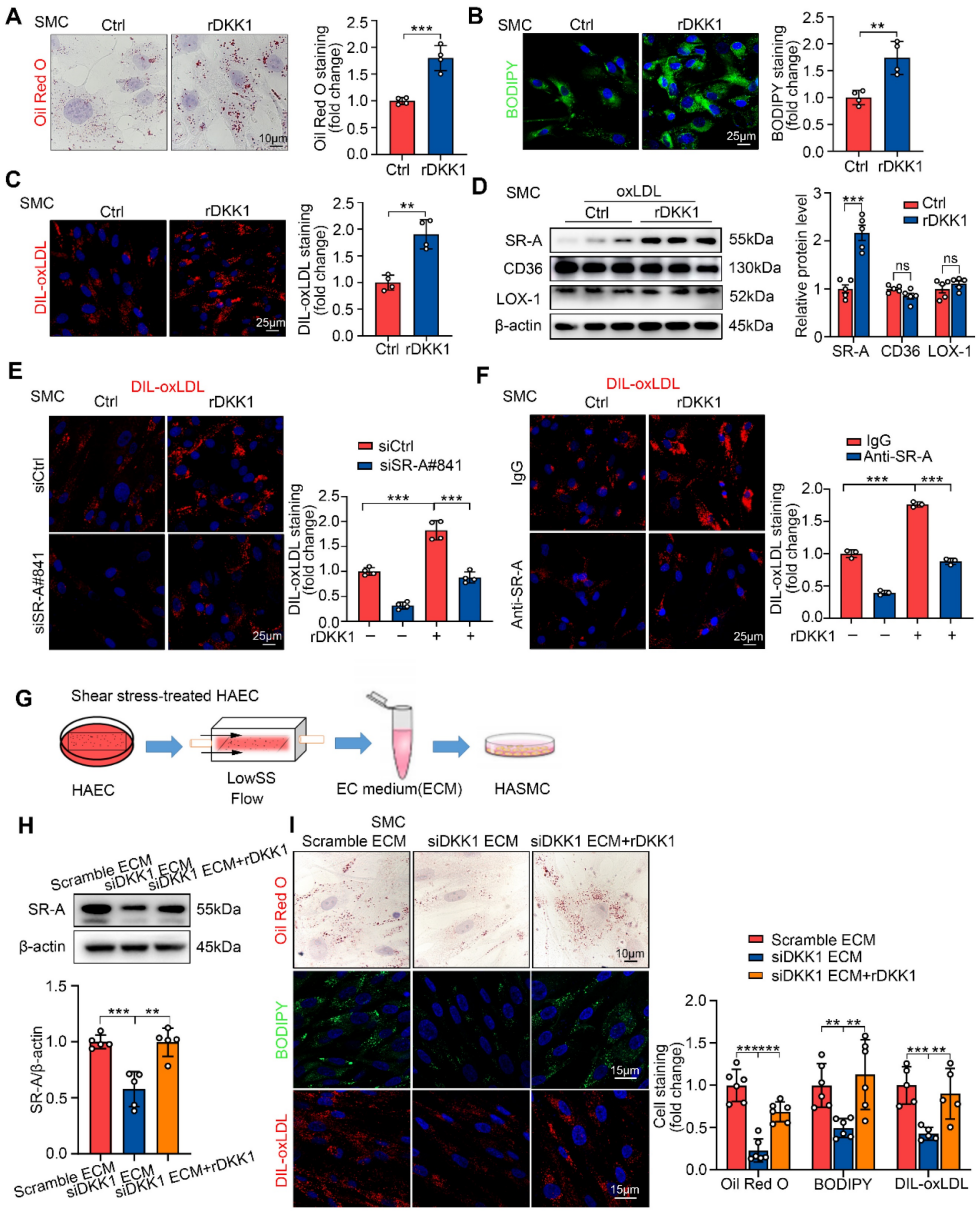
** Exogenous DKK1 promotes lipid uptake in SMCs via upregulation of SR-A. (A-C)** Representative images and quantification of SMCs labeled by Oil Red O (A), BODIPY493/503 (B), or DIL-oxLDL (C). SMCs were treated with rDKK1 (100 ng/mL) or PBS for 24 h (n = 4). **(D)** Protein expression and quantification of SR-A, CD36, and LOX-1 in SMCs incubated with or without rDKK1 under oxLDL stimulation (n = 5). **(E)** Representative images of DIL-oxLDL uptake in SMCs transfected with siCtrl or siSR-A with or without rDKK1 incubation (n = 4). **(F)** Representative images of DIL-oxLDL uptake in SMCs incubated with SR-A neutralizing antibody (10 μg/mL) or IgG with or without rDKK1 (n = 3). **(G)** Schematic illustration of the setup of the ECs model subjected to shear stress and the procedure how the SMCs were incubated with shear stress treated-endothelial medium (ECM). **(H)** Protein expression and quantification of SR-A in SMCs incubated with shear stress treated-endothelial medium (ECM) under rDKK1 or not (n = 5). **(I)** Oil Red O (upper panel, n=6), BODIPY493/503 (middle panel, n=6), DIL-oxLDL (lower panel, n=5) staining and quantification in SMCs incubated with shear stress treated-endothelial medium (ECM) under rDKK1 or not. ^**^p < 0.01, ^***^p < 0.001, ns not significant. Data are shown as the Mean ± SD. *p* values were calculated by Two-tailed unpaired Student's *t*-test or One-way ANOVA followed by Tukey's multiple comparison test.

To validate the effect of exogenous DKK1 on lipid deposition in SMCs, SMCs were cultured independently and stimulated with recombinant DKK1 protein (rDKK1, 100 ng/mL) for 24 h followed by oxLDL treatment. rDKK1 significantly promoted intracellular lipid deposition, DIL-oxLDL uptake and SR-A expression in SMCs (Figure 3A-D). Furthermore, rDKK1-mediated DIL-oxLDL uptake in SMCs was significantly inhibited by the siRNA-mediated knockdown of SR-A (Figure 3E) or SR-A-neutralizing antibody (Figure 3F), indicating that DKK1 promotes lipid uptake in SMCs by upregulating SR-A. The knock-down efficiency of SR-A in SMCs was shown in Figure S4A. Furthermore, we knocked down DKK1 in ECs prior to LowsSS treatment for 24 h and subsequently collected the conditioned medium from shear stress-treated ECs to incubate SMCs (Figure 3G). The results demonstrated a significant inhibition of lipid deposition, lipid uptake, and SR-A expression in SMCs upon knockdown of DKK1 in ECs. However, the reduced levels of lipid deposition, lipid uptake, and SR-A expression were restored after local replenishment of rDKK1 in SMCs (Figure 3H, 3I).

DKK1 is a secretory glycoprotein that exerts paracrine effects by interacting with the DKK1 receptors CKAP4 and LRP6 17, 18 which are abundantly expressed on the surface of SMCs (Figure 1D). To determine the receptor through which DKK1 signals to promote SMC-derived foam cell formation, rDKK1 was used to stimulate SMCs with either CKAP4 or LRP6 knockdown. The interference efficiencies of CKAP4 and LRP6 in SMCs are shown in Figure S4B, S4C. Our data demonstrated that both CKAP4 and LRP6 mediated the effects of DKK1 on lipid deposition, lipid uptake (Figure S4D-G) and SR-A expression (Figure S4H, S4I) in SMCs. As previously reported, DKK1 simultaneously binds to both CKAP4 and LRP6 to form a ternary complex 18. Consistent with this report 26, our findings showed that CKAP4 and LRP6 efficiently co-immunoprecipitated under stimulation of rDKK1 in SMCs (Figure S4J).

Collectively, these findings suggest that DKK1 signals CKAP4 and LRP6 to promote oxLDL-induced foam cell formation by upregulating SR-A expression in SMCs.

### Endothelial-specific knock-out of DKK1 ameliorates AS and lipid accumulation in SMCs within plaques

**Figure 4:**
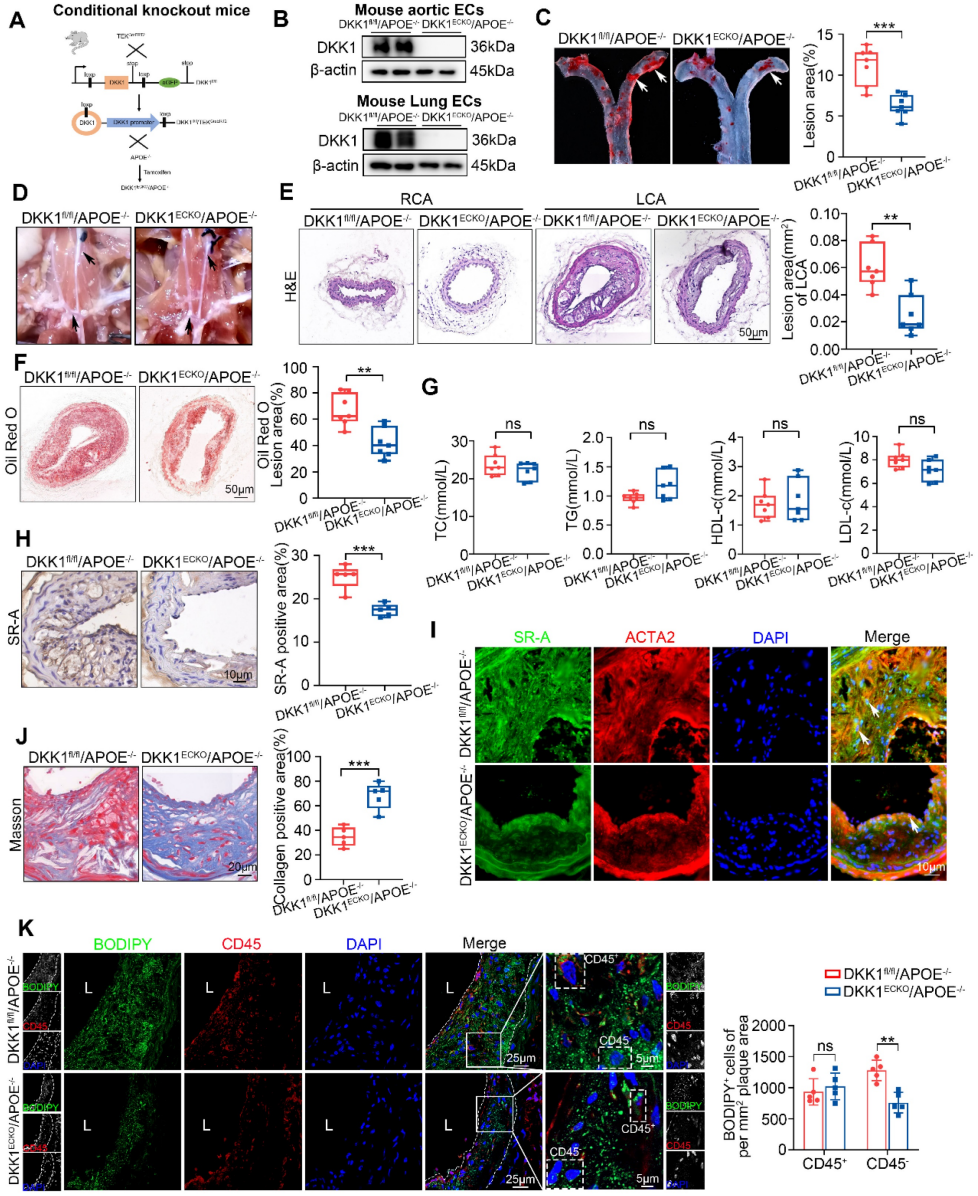
** Endothelial-specific knock-out of DKK1 ameliorated AS and lipid accumulation in SMCs within plaques. (A)** Model of EC-specific DKK1 knockout in APOE^-/-^ mice was established.** (B)** Western blot for DKK1 in primary aortic or lung ECs from DKK1^fl/fl^/APOE^-/-^ and DKK1^ECKO^/APOE^-/-^ mice. **(C)** Oil Red O staining of aortas from male DKK1^fl/fl^/APOE^-/-^ and DKK1^ECKO^/APOE^-/-^ mice fed a high-fat diet (HFD) for 8 weeks following the partial ligation of the left common carotid artery (LCA) (n = 7). **(D)** Representative gross photographic images of atherosclerotic plaques in the aortic arch and common carotid arteries (black arrows) from DKK1^fl/fl^/APOE^-/-^ and DKK1^ECKO^/APOE^-/-^ mice. **(E)** Hematoxylin and eosin (H&E) staining and cross-sectional analysis of plaque area in the LCA (n = 7). The un-ligated right common carotid artery (RCA) was used as the sham group. **(F)** Oil Red O staining and cross-sectional analysis of atherosclerotic lesions in the LCA (n = 7). **(G)** Serum concentrations of TC, TG, HDL-c and LDL-c were determined using Beckman automatic biochemical analyzer (n = 7).** (H)** Immunohistochemical detection of SR-A in carotid plaques (n = 5). **(I)** Double immunofluorescence staining for SR-A (green) and ACTA2 (red) in carotid plaques from the indicated groups. In the merged images, white arrows indicate overlap of SR-A and ACTA2 staining. **(J)** Quantification of collagenous fibers by Masson staining in DKK1^fl/fl^/APOE^-/-^ and DKK1^ECKO^/APOE^-/-^ mice (n = 5). **(K)** Representative immunofluorescence staining of CD45 (red) and BODIPY493/503 (green) in atherosclerotic lesions of the LCA from DKK1^fl/fl^/APOE^-/-^ and DKK1^ECKO^/APOE^-/-^ mice (n = 5). ^**^p < 0.01, ^***^p < 0.001, ns not significant. Data are shown as the Mean ± SD. *p* values were calculated by two-tailed unpaired Student's *t*-test.

EC-specific DKK1-knockout (DKK1^ECKO^) mice were crossed with APOE^-/-^ mice to generate DKK1^ECKO^/APOE^-/-^ mice to elucidate the role of endothelial DKK1 in SMC-derived foam cell formation and AS development (Figure 4A; Figure S5A). The deletion of the DKK1 gene in mouse ECs was validated by western blot and immunofluorescence staining (Figure 4B; Figure S5B-E). The LCA was partially ligated to induce disturbed shear stress in arteries of DKK1^ECKO^/APOE^-/-^ and DKK1^fl/fl^/APOE^-/-^ mice, who were then fed a HFD for 8 weeks. DKK1^ECKO^/APOE^-/-^ mice exhibited markedly fewer atherosclerotic lesions than DKK1^fl/fl^/APOE^-/-^ mice (Figure 4C-F), without affecting the serum lipid profile (Figure 4G; Table S1). In addition, SR-A expression within plaques was significantly downregulated in DKK1^ECKO^/APOE^-/-^ mice, particularly in SMCs (Figure 4H, 4I). Moreover, the collagen content was substantially higher in DKK1^ECKO^/APOE^-/-^ mice than in control mice, as indicated by Masson staining (Figure 4J). The leukocyte marker CD45 and the lipid-specific fluorescent dye BODIPY493/503 were then used to distinguish foam cells derived from non-leukocytes (predominantly SMC-derived), as determined by CD45^-^/BODIPY493/503^+^, and foam cells derived from leukocytes, as determined by CD45^+^/BODIPY493/503^+^ within the plaques. The results showed that EC-specific knockout of DKK1 reduced lipid deposition in SMCs within plaques, as shown by decreased numbers of CD45^-^/BODIPY493/503^+^ cells per mm^2^ of plaque area; however, there was no significant effect on the number of CD45^+^/BODIPY493/503^+^ cells (Figure 4K). We used a variety of markers including ACTA2/α-SMA, MYH11, and TAGLN to label differentiated SMCs, and PDGFRB/PDGFRβ to label de-differentiated SMCs within plaques 28. We found that DKK1 deficiency in ECs significantly reduced lipid deposition in both ACTA2^+^ and PDGFRβ^+^ cells per mm^2^ plaque aera (Figure S6A, S6B). This was confirmed by the co-staining of MYH11/BODIPY493/503 and TAGLN/BODIPY493/503 in plaques (Figure S6C, S6D).

These data demonstrate that DKK1 deficiency in ECs ameliorates the atherosclerotic plaque burden and reduces the size of the lipid core and the number of SMC-derived foam cells within atherosclerotic plaques.

### Endothelial-specific overexpression of DKK1 exacerbates AS and lipid accumulation in SMCs within plaques

**Figure 5:**
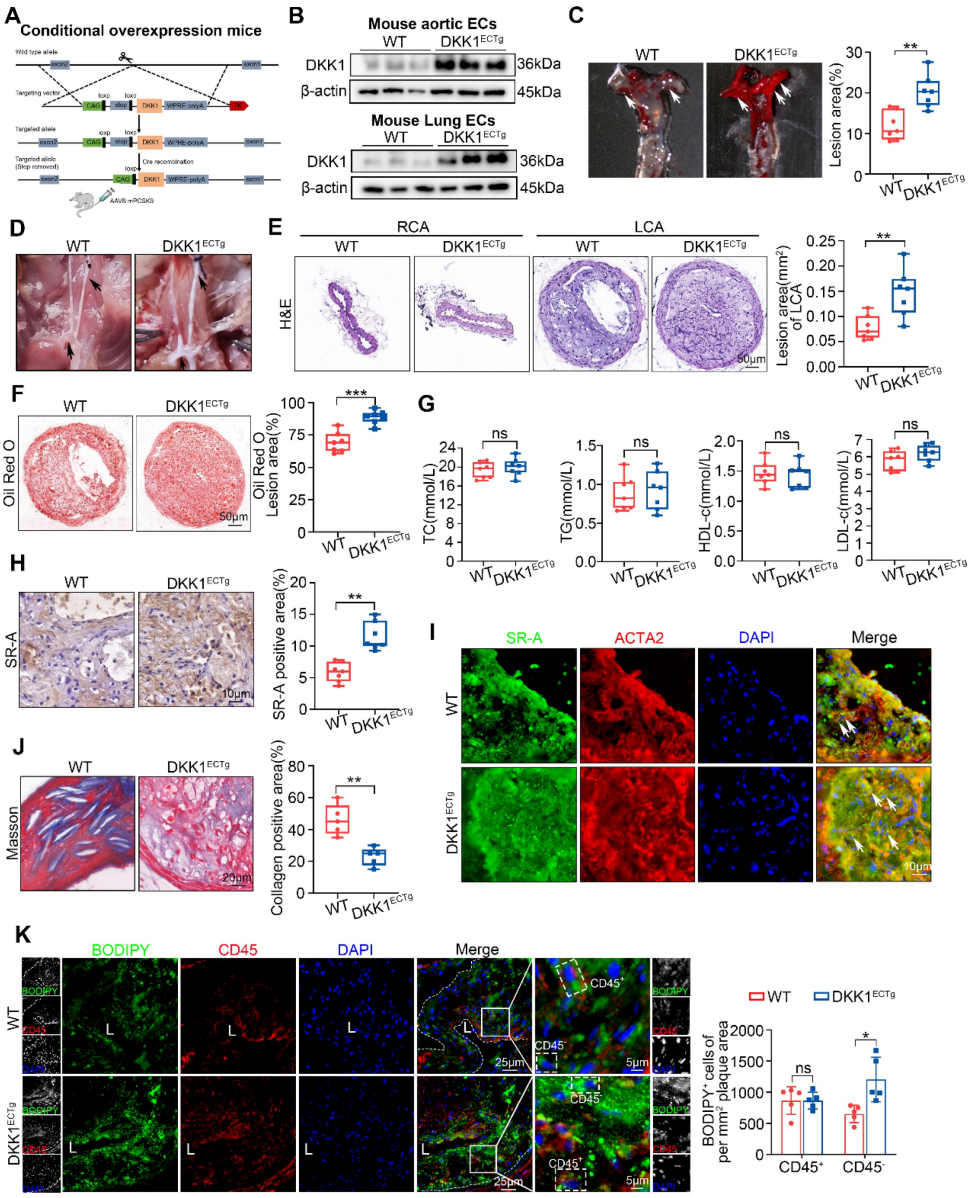
** Endothelial-specific overexpression of DKK1 exacerbates AS and lipid accumulation in SMCs within plaques. (A)** A flow chart illustrates the procedure of DKK1 transgene in ECs conditionally. Wild Type (WT) and DKK1^ECTg^ mice (C57BL/6J background) were injected with adeno-associated virus 8 (AAV8)-mPCSK9, and subjected to partial carotid ligation followed by a HFD for 8 weeks. **(B)** Representative western blot of DKK1 in primary mouse aortic or primary mouse lung ECs from WT and DKK1^ECTg^ mice. **(C)** Representative images and analysis of the Oil Red O-stained AA, especially the inner curvature (white arrows) of WT and DKK1^ECTg^ mice (n = 7).** (D)** Gross photographic images of a carotid plaque, AA, and branches (black arrows) from WT and DKK1^ECTg^ mice. **(E, F)** Representative images of frozen sections of the left common carotid plaque isolated from WT and DKK1^ECTg^ mice. The sections were stained with H&E (E) and Oil Red O (F) (n = 7). The un-ligated RCA was used as the sham group. **(G)** Serum levels of TC, TG, HDL-c, and LDL-c in WT and DKK1^ECTg^ mice after being fed a HFD for 8 weeks (n = 7). **(H)** Immunohistochemical detection and quantification of SR-A (n = 7). **(I)** Double immunofluorescence staining for SR-A (green) and ACTA2 (red) in the carotid plaque from WT and DKK1^ECTg^ mice. White arrows indicate cells that are positive for both SR-A and ACTA2. **(J)** Representative images of a Masson-stained carotid plaque from the indicated group. Collagenous fibers were quantified (n = 5).** (K)** Representative images of carotid plaque sections obtained from WT and DKK1^ECTg^ mice stained with CD45 and BODIPY493/503 (n = 5). ^**^p < 0.01, ^***^p < 0.001, ns not significant. Data are shown as the Mean ± SD. *p* values were calculated by two-tailed unpaired Student's *t*-test.

To further confirm the role of endothelial DKK1 in AS, we established EC-specific DKK1-transgenic mice (DKK1^ECTg^) (Figure 5A; Figure S7A). The overexpression of the DKK1 gene in mouse ECs was validated by western blot and immunofluorescence staining (Figure 5B; Figure S7B-D). Next, the LCA in DKK1^ECTg^ and WT mice was partially ligated. AS was induced in mice via tail vein injection of AAV8 encoding the Asp374-to-Tyr mutant version of PCSK9 (PCSK9DY), followed by feeding HFD diet for 8 weeks 29-31. Compared with WT mice, DKK1^ECTg^ mice developed more atherosclerotic lesions and lipid content (Figure 5C-F), without affecting the serum lipid profile (Figure 5G; Table S2). Furthermore, the expression of SR-A was significantly upregulated within the plaques of DKK1^ECTg^ mice (Figure 5H), particularly in SMCs within the plaques, as verified by double immunofluorescence staining for SR-A (green) and ACTA2 (red) (Figure 5I). In addition, collagen content was substantially lower in DKK1^ECTg^ mice compared with WT mice, as verified by Masson staining (Figure 5J). Next, we identified foam cells within the plaques derived from SMCs as being CD45-negative (CD45^-^) and BODIPY493/503-positive (BODIPY493/503^+^). Figure 5K shows that EC-specific overexpression of DKK1 promotes lipid deposition in SMCs within plaques, as indicated by an increased number of CD45^-^/BDDIPY493/503^+^ cells per mm^2^ of plaque area, while having no significant effects on the number of CD45^+^/BODIPY493/503^+^ cells. In addition, using double immunofluorescence staining with specific markers for differentiated (ACTA2, MYH11, and TAGLN) and de-differentiated (PDGFRβ) SMCs, together with the lipid-specific fluorescent dye BODIPY493/503, we observed significantly higher levels of lipid deposition in SMCs within plaques from DKK1^ECTg^ mice compared to those from WT mice (Figure S8A-D).

These data demonstrate that DKK1 overexpression in ECs increases the atherosclerotic plaque burden and the number of SMC-derived foam cells.

### LowSS-induced endothelial DKK1 facilitates SR-A stabilization in co-cultured SMCs by inhibiting the proteasomal degradation pathway

**Figure 6:**
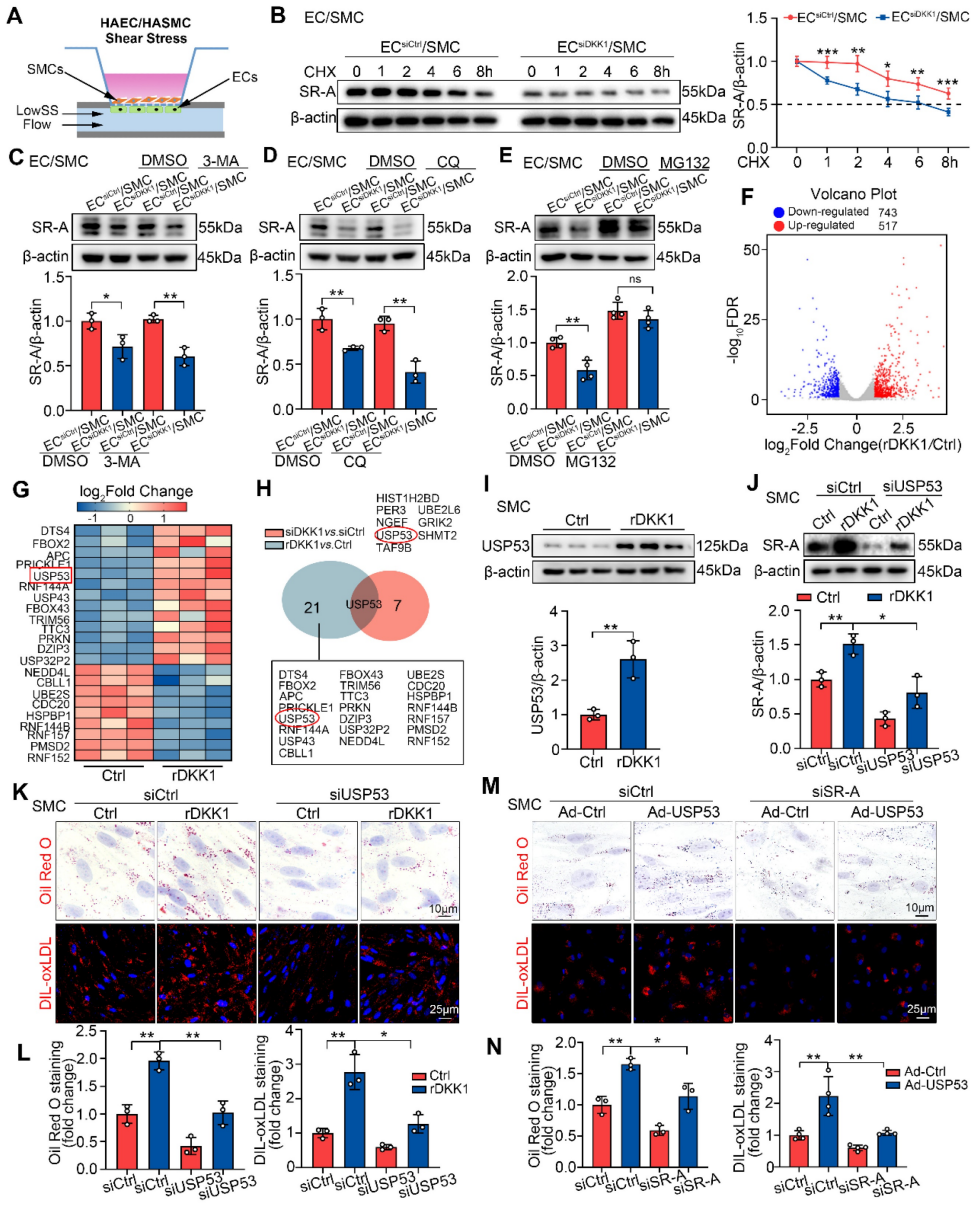
** LowSS-induced endothelial DKK1 facilitates SR-A stabilization in co-cultured SMCs by inhibiting the proteasomal degradation pathway mediated by USP53. (A)** Schematic diagram of an EC/SMC co-culture flow system. ECs were stimulated with LowSS. **(B)** The effect of CHX (10 μg/mL) on the half-life of SR-A in HASMCs co-cultured with HAECs transfected with siCtrl or siDKK1 under LowSS conditions (n = 5).** (C-E)** The impact of 3-MA (10 mM for 12 h) (C, n = 3), CQ (10 μM for 12h) (D, n = 3), or MG132 (1 μM for 12 h) (E, n = 4) on the expression of SR-A in HASMCs co-cultured with HAECs transfected with siCtrl or siDKK1 under LowSS conditions. **(F, G)** HASMCs incubated with rDKK1 for 24 h were analyzed by RNA sequencing. Differentially expressed genes (DEGs) visualized using a volcano plot (F). Selected genes involved in the ubiquitin-proteasome system are visualized as a heatmap (G). **(H)** RNA sequencing results for HASMCs transfected with siDKK1 for 48 h are presented in Figure S9C, S9D. USP53 is the only ubiquitin-related gene present in both DEGs (red circle).** (I)** Protein expression of USP53 in HASMCs following incubation with rDKK1 for 24 h (n = 3). **(J)** The expression of SR-A in HASMCs transfected with siUSP53 with or without rDKK1 (n = 3). **(K, L)** Oil Red O (upper panel, n = 3) and DIL-oxLDL (lower panel, n = 3) staining of HASMCs transfected with siUSP53 with or without rDKK1. **(M, N)** Oil Red O (upper panel, n = 3) and DIL-oxLDL (lower panel, n = 4) staining of HASMCs transfected with Ad-USP53 with or without siSR-A. ^*^p < 0.05, ^**^p < 0.01, ^***^p < 0.001, ns not significant. Data are shown as the Mean ± SD. *p* values were calculated by Two-tailed unpaired Student's *t*-test or One way ANOVA followed by Tukey's multiple comparison test.

We then investigated the mechanism by which endothelial DKK1 regulates SR-A expression in co-cultured SMCs. DKK1 increased SR-A protein levels in SMCs (Figure 3D), but had no effect on SR-A mRNA levels (Figure S9A). ECs transfected with Control-siRNA (EC^siCtrl^) or DKK1-siRNA (EC^siDKK1^) were co-cultured with SMCs and then stimulated with LowSS for 24 h (Figure 6A). We found that DKK1 deficiency in ECs did not alter the mRNA levels of SR-A in co-cultured SMCs (Figure S9B), indicating that LowSS-induced endothelial DKK1 regulates SR-A expression in co-cultured SMCs at the post-translational level. Using the protein synthesis inhibitor cycloheximide (CHX), we found that the half-life of SR-A was significantly shortened in SMCs co-cultured with EC^siDKK1^ under LowSS stimulation (Figure 6B). This indicates that DKK1 deficiency in ECs can accelerate the degradation of SR-A protein in co-cultured SMCs. Proteasomes, lysosomes, and autophagy are three principal pathways of intracellular protein degradation. To explore the specific mechanism by which DKK1 regulates SR-A protein degradation, SMCs co-cultured with EC^siCtrl^ or EC^siDKK1^ under LowSS conditions were treated with proteasome, lysosome, or autophagy inhibitors. As shown in Figure 6C and 6D, neither 3-MA, a potent lysosomal inhibitor, nor chloroquine (CQ), an autophagy inhibitor, influenced the SR-A protein level or reversed the reduction of SR-A in SMCs caused by DKK1-knockdown in ECs under LowSS. However, inhibition of the proteasomal degradation pathway by MG132, a potent 26S proteasome inhibitor, significantly upregulated SR-A protein levels and completely rescued the decrease in SR-A levels in SMCs co-cultured with EC^siDKK1^ under LowSS (Figure 6E). These findings suggest that LowSS-induced endothelial DKK1 may play a crucial role in regulating the proteasomal degradation of SR-A in co-cultured SMCs.

### DKK1 inhibits proteasomal degradation of SR-A by upregulating USP53

To identify the target proteins involved in the DKK1-mediated proteasomal degradation of SR-A, SMCs treated with rDKK1 or DKK1 siRNA knockdown were analyzed by RNA sequencing (RNA-seq). Among the altered genes in rDKK1 group, 22 genes were related to the ubiquitin proteasome pathway (UPP) (Figure 6F, 6G), while the DKK1-knockdown group, eight genes related to the UPP system (Figure S9C, S9D). Among the genes regulated by rDKK1 and those altered by DKK1 knockdown, USP53 was the only one present in both groups (Figure 6H). Next, we confirmed USP53 mRNA and protein expression in SMCs treated with rDKK1 or DKK1 knockdown (Figure 6I; Figure S9E-G). Double immunofluorescence staining with ACTA2 (red) and USP53 (green) in DKK1^ECKO^/APOE^-/-^ and DKK1^ECTg^ mice demonstrated that USP53 in SMCs within plaques was affected by endothelial DKK1 (Figure S9H, S9I). In addition, by using co-culture system, we found that knockdown of endothelial DKK1 inhibited the LowSS-induced upregulation of USP53 in co-cultured SMCs (Figure S9J).

Our *in vivo* and *in vitro* data showed that USP53 is a vital target which may play an important role in AS (Figure S10A-G). The interference efficiency of USP53-siRNA, and overexpression efficiency of the adenovirus in SMCs are shown in Figure S10H. Furthermore, SR-A expression was correlated with USP53 levels (Figure S10I, S10J). Importantly, USP53 knockdown significantly attenuated the rDKK1-induced upregulation of SR-A expression in SMCs (Figure 6J), and reduced the rDKK1-induced lipid accumulation and DIL-oxLDL uptake (Figure 6K, 6L). In addition, SR-A knockdown reduced the stimulatory effect of USP53 on SMC-derived foam cell formation (Figure 6M, 6N). In summary, DKK1 inhibits proteasomal degradation of SR-A and promotes lipid deposition in SMCs by targeting USP53.

Next, we elucidated the mechanism through which USP53 was regulated by DKK1. We observed significantly increased USP53 transcription in rDKK1-treated SMCs previously (Figure S9E), implying that USP53 expression may be regulated by DKK1 at the transcription level primarily. Previous studies show that there are a few studies on the upstream regulatory mechanism of USP53 32, 33. Among them, the transcription factor CREB raised our interest, which was reported to be positively associated with hepatocyte lipid accumulation 34, 35. Immunoblot analysis verified that the expression of CREB was upregulated by rDKK1 (Figure S11A). Furthermore, CREB knockdown resulted in reduced USP53 mRNA and protein levels in SMCs (Figure S11B, S11C). Furthermore, the upregulation of USP53 induced by rDKK1 was markedly reversed by CREB siRNA (Figure S11D). These findings were validated by chromatin immunoprecipitation (ChIP) assay.

As expected, CREB specifically bound to the USP53 promoter, which could be enhanced by rDKK1 (Figure S11E). To explore the mechanism through which CREB was regulated by DKK1, a Kyoto Encyclopedia of Genes and Genomes (KEGG) pathway analysis was performed (Figure S11F), focusing on the PI3K-Akt pathway, which is related to the functions of DKK1 18. We found that rDKK1 promoted Akt phosphorylation in SMCs significantly (Figure S11G). In addition, the PI3K inhibitor LY294002 significantly attenuated rDKK1-induced upregulation of CREB protein expression (Figure S11H). Taken together, these data suggest that DKK1 regulated USP53 transcription through PI3K/AKT-mediated binding of CREB to the USP53 promoter.

### USP53 stabilizes SR-A protein by inhibiting K48-linked polyubiquitination

**Figure 7:**
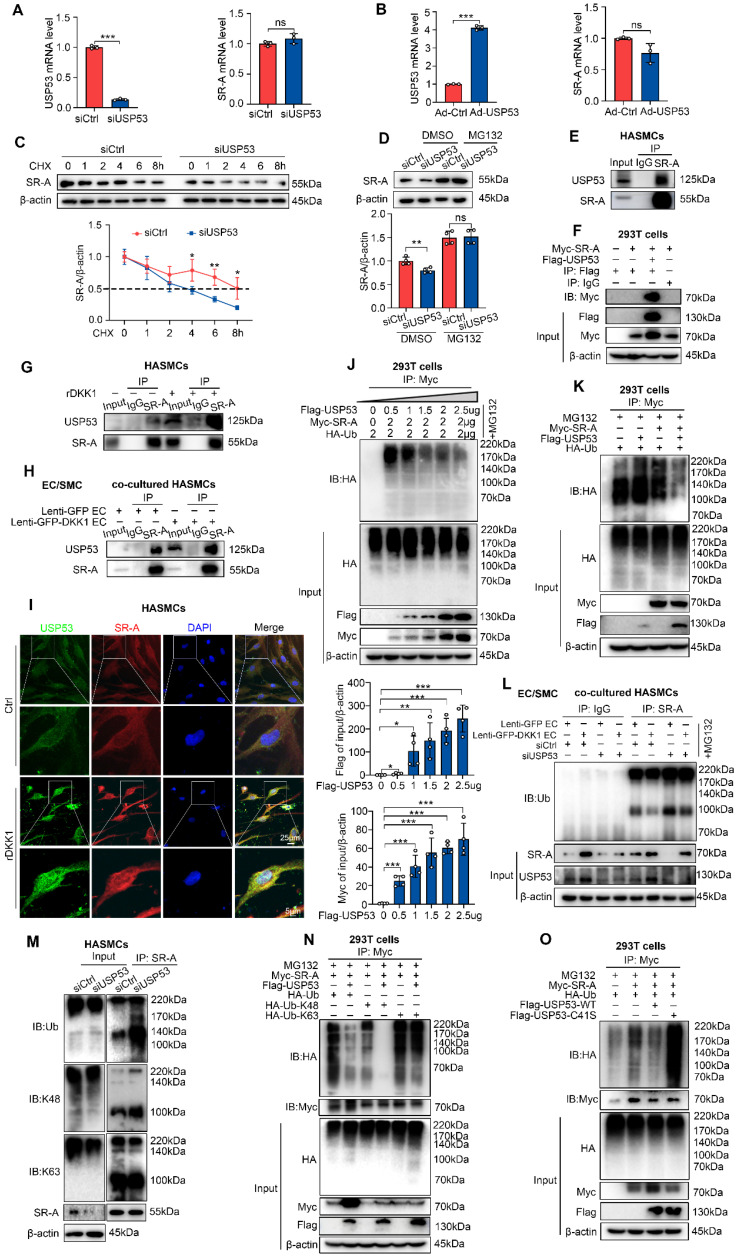
** USP53 stabilizes SR-A protein by inhibiting K48-linked polyubiquitination. (A, B)** mRNA levels of USP53 (left panel) and SR-A (right panel) in HASMCs transfected with siUSP53 (A) or Ad-USP53 (B) (n = 3 each). **(C)** Knockdown of USP53 in HASMCs followed by CHX (10 μg/mL) treatment for the indicated duration. SR-A protein expression was analyzed by western blot (n = 4). **(D)** Western blot for SR-A in HASMCs incubated with MG132 or DMSO for 12 h with or without siUSP53 transfection (n = 4). **(E)** Co-IP assay to investigate the endogenous interaction between USP53 and SR-A in HASMCs. **(F)** Co-IP analysis investigating the association of USP53 with SR-A in HEK293T cells co-transfected with Myc-tagged SR-A and Flag-tagged USP53 plasmids. **(G)** Co-IP assay investigating the endogenous interaction between USP53 and SR-A in HASMCs after rDKK1 incubation. **(H)** Co-IP analysis investigating the association of USP53 with SR-A in HASMCs co-cultured with Lenti-GFP HAECs or Lenti-GFP-DKK1 HAECs. **(I)** Representative images of immunofluorescence staining showing the co-localization of USP53 (green) and SR-A (red) in HASMCs with or without rDKK1 incubation.** (J)** Co-IP assay investigating the Ub levels of SR-A in HEK293T cells transfected with a concentration gradient of Flag-tagged USP53, 2 μg Myc-tagged SR-A, and 2 μg HA-tagged Ub (n = 4). **(K)** Co-IP analysis for SR-A ubiquitination in HEK293T cells transfected with plasmids expressing Myc-tagged SR-A, Flag-tagged USP53, and HA-tagged Ub. **(L)** Co-IP assay for SR-A ubiquitination in siCtrl or siUSP53-transfected HASMCs co-cultured with HAECs transfected with Lenti-GFP or Lenti-GFP-DKK1. **(M)** Co-IP analysis for endogenous USP53 poly-ubiquitination in HASMCs transfected with siCtrl or siUSP53. **(N)** HEK293T cells were co-transfected with plasmids encoding Myc-tagged SR-A, HA-tagged Ub, HA-tagged Ub-K48, HA-tagged Ub-K63, and Flag-tagged USP53. Cells were then exposed to MG132 to inhibit proteasomal degradation. SR-A ubiquitination was assessed by Co-IP assay using a Myc antibody. **(O)** Co-IP analysis was performed to investigate the ubiquitination of Myc-tagged SR-A in HEK293T cells transfected with Flag-tagged USP53 WT and Flag-tagged mutant USP53 C41S plasmids. ^*^p < 0.05, ^**^p < 0.01, ^***^p < 0.001, ns not significant. Data are shown as the Mean ± SD. The results were analyzed by Two-tailed unpaired Student's *t*-test or Multiple linear mixed effects modelling or One way ANOVA followed by Tukey's multiple comparison test.

Mechanistically, we found that neither siUSP53 nor Ad-USP53 affected SR-A mRNA level (Figure 7A, 7B). In the presence of CHX, USP53 knockdown significantly shortened the half-life of SR-A in SMCs (Figure 7C), indicating that USP53 may regulate SR-A expression at the post-translational level. Furthermore, the siUSP53-induced downregulation of SR-A in SMCs was reversed by MG132 (Figure 7D), demonstrating that ubiquitin-proteasome pathway contributes to the upregulation of SR-A by USP53.

To explore whether USP53 interacts with SR-A, co-immunoprecipitation (Co-IP) and immunoblotting (IB) experiments were performed using HASMCs or HEK293T. The results showed that USP53 and SR-A efficiently co-immunoprecipitated (Figure 7E, 7F). Additionally, the interaction between USP53 and SR-A in HASMCs was enhanced by exogenous rDKK1 treatment (Figure 7G) or co-culturing with ECs overexpressing DKK1 (Lenti-GFP-DKK1 EC, Figure 7H). These findings were validated by double immunofluorescence staining for USP53 (green) and SR-A (red) in HASMCs (Figure 7I).

To test whether USP53 modulates SR-A ubiquitination, HEK293T cells were co-transfected with HA-tagged ubiquitin (Ub), Myc-tagged SR-A, and dose-dependent Flag-tagged USP53 plasmids. We found that USP53 markedly downregulated Ub levels and inhibited SR-A degradation (Figure 7J, 7K). In addition, higher levels of total SR-A ubiquitination in USP53-deficient HASMCs compared with that in control cells (Figure 7M). In addition, by using co-culturing HASMCs transfected with USP53-siRNA and HAECs transfected with Lenti-GFP-DKK1, we found that USP53 knockdown reversed the downregulation of SR-A ubiquitination induced by endothelial DKK1 in co-cultured HASMCs (Figure 7L). Moreover, western blot analysis showed that the K48-linked but not K63-linked ubiquitin chain was the major form removed by USP53 of SR-A (Figure 7M, 7N).

Given that USP53 is a deubiquitinating enzyme (DUB), we next examined whether its impact on SR-A ubiquitination depends on its enzymatic activity. We constructed a C41S mutant (mutation of cysteine to serine at position 41) which lacks deubiquitination activity and co-transfected Myc-tagged SR-A with Flag-tagged USP53 WT or its enzyme inactive mutant C41S into HEK293T cells. We observed that the deubiquitination effect of USP53 on SR-A was abolished by mutant C41S (Figure 7O), indicating that USP53 enzymatic activity was indispensable for the deubiquitination of SR-A.

These results demonstrate that USP53 interacts with SR-A, which can be enhanced by endothelial DKK1. USP53 stabilizes SR-A by inhibiting K48-linked polyubiquitination in SMCs.

### SMC-specific USP53 overexpression reversed the alleviation of atherosclerotic plaque burden and lipid accumulation in SMCs within plaques from DKK1^ECKO^/APOE^-/-^ mice

**Figure 8:**
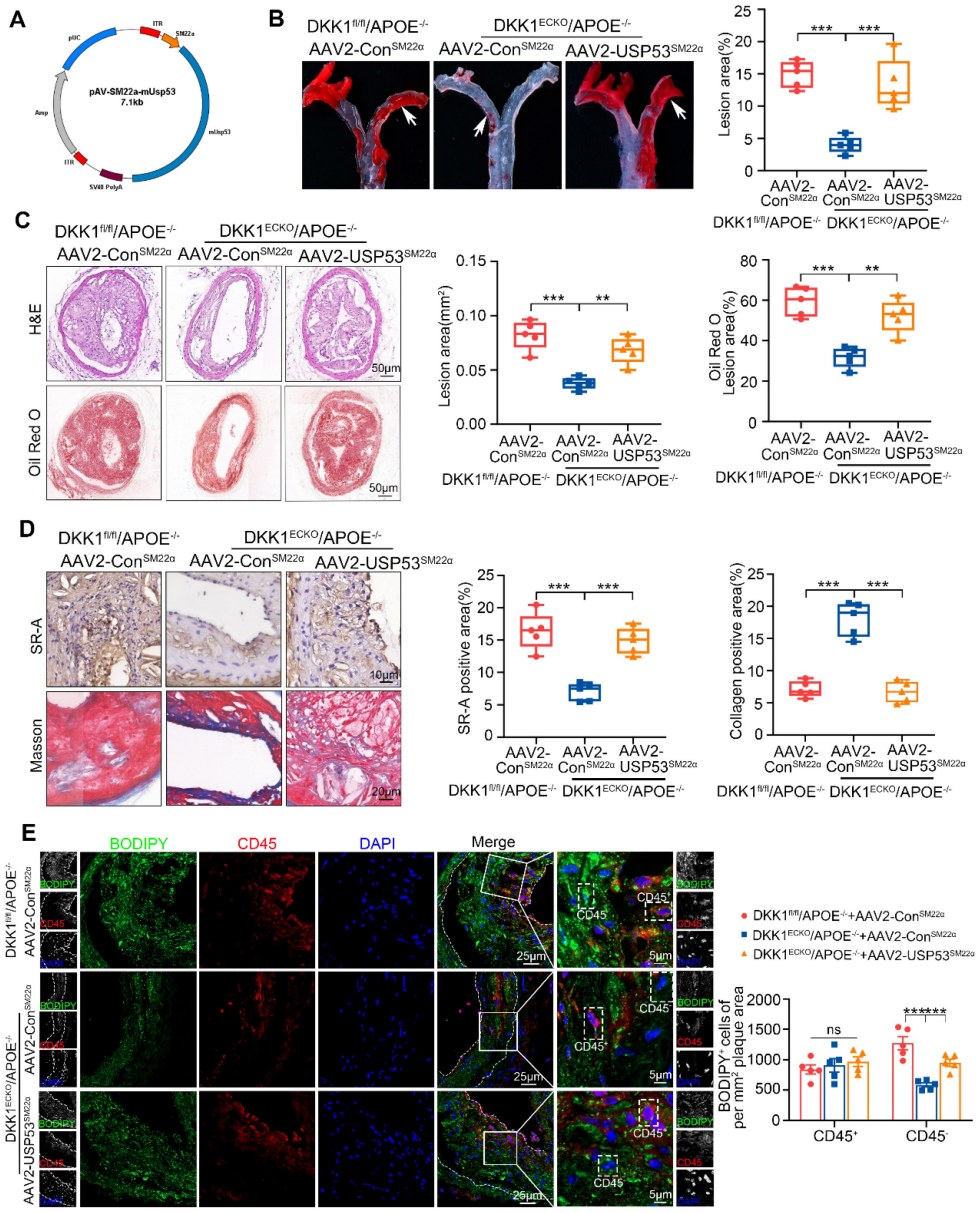
** SMC-specific overexpression of USP53 reversed the alleviation of atherosclerotic plaque burden and lipid accumulation in SMCs within plaques from DKK1^ECKO^/APOE^-/-^ mice. (A)** Schematic diagram showing AAV2-SM22α-USP53 overexpression in SMCs. **(B)** Representative images of Oil Red O staining showing the total AA *en face,* especially the inner curvature (white arrows), obtained from three groups of mice (DKK1^fl/fl^/APOE^-/-^+AAV2-Con^SM22α^ group, DKK1^ECKO^/APOE^-/-^+AAV2-Con^SM22α^ group, DKK1^ECKO^/APOE^-/-^+AAV2-USP53^SM22α^ group) (n = 5 per group).** (C)** H&E (upper panel) and Oil Red O (lower panel) staining of LCA plaques and analysis of the indicated groups (n = 5 per group). **(D)** Immunohistochemical staining for SR-A (upper panel), Masson staining (lower panel) within plaques, and analysis of the indicated groups (n = 5 per group). **(E)** Immunofluorescence staining for CD45 combined with BODIPY493/503 staining (green fluorescent particles) was performed to assess lipid deposition in SMCs within LCA plaques in each group (n = 5 per group). ^**^p < 0.01, ^***^p < 0.001, ns not significant. Data are shown as the mean ± SD. One way ANOVA followed by Tukey's multiple comparison test was used for statistical analysis.

To confirm the mediating role of USP53 in the effect of endothelial DKK1 on AS and SMC-derived foam cell formation *in vivo*, DKK1^ECKO^/APOE^-/-^ mice were injected with recombinant AAV2 vectors that overexpressed SMCs-specific USP53 (AAV2-SM22α-USP53) or Control vector (AAV2-SM22α-Con) for 4 weeks (Figure 8A). DKK1^fl/fl^/APOE^-/-^ mice injected with AAV2-SM22α-Con were used as controls. These mice then underwent partial carotid ligation and received a HFD for 8 weeks. Elevated USP53 expression was detected in the aortas and primary aortic SMCs of AAV2-SM22α-USP53-treated mice (Figure S12A). Correspondingly, double immunofluorescence staining showed that USP53 expression was specifically upregulated in the tunica media of aortas (Figure S12B). Under treatment of AAV2-SM22α-Con, DKK1^ECKO^/APOE^-/-^ mice exhibited a significant reduction in atherosclerotic plaque burden, lesion area, lipid deposition, SR-A expression within plaques, and an increase in collagen content within plaques compared with DKK1^fl/fl^/APOE^-/-^ mice. However, overexpression of AAV2-SM22α-USP53 abrogated these observed alleviations (Figure 8B-D). To determine whether USP53 mediates the effects of endothelial DKK1 on SMC-derived foam cell formation *in vivo*, the neutral lipid stain BODIPY493/503 and the leukocyte marker CD45 were visualized by fluorescence microscopy to distinguish between foam cell populations derived from leukocytes (macrophage-derived) and non-leukocytes (SMC-derived) in the atherosclerotic lesions of the LCA.

The CD45^-^/BODIPY493/503^+^ cell population represented the SMC-derived foam cells. We found that SMC-specific overexpression of USP53 in DKK1^ECKO^/APOE^-/-^ mice aggravated lipid deposition in SMCs within plaques, as indicated by an increased number of CD45^-^/BODIPY493/503^+^ cells per mm^2^ of the plaque area, but had no significant effect on the number of CD45^+^/BODIPY493/503^+^ cells (Figure 8E). Additionally, immunofluorescence staining was performed using the SMC marker ACTA2 and PDGFRβ together with BODIPY493/503. The results indicate that the inhibitory effect of DKK1 knockout in ECs on the formation of SMC foam cells was significantly reversed by USP53-specific SMC overexpression (Figure S13). In summary, DKK1 deficiency in ECs attenuates the development of AS and inhibits SMC-derived foam cell formation in a USP53-dependent manner.

## Discussion

In the present study, we demonstrated that endothelial-secreted DKK1, induced through pathological shear stress, promotes foam cell formation in co-cultured SMCs via deubiquitination of SR-A by USP53 (summarized in the Graphic Abstract). This conclusion is supported by findings obtained through *in vivo* and *in vitro* experiments: (1) the protein level of DKK1 was upregulated in both ECs and SMCs in a co-culture model under low shear stress; (2) the upregulation of DKK1 protein in SMCs was secondary to the elevated transcription of DKK1 in ECs rather than SMCs themselves in the co-culture model under low shear stress, indicating that SMCs could take up low shear stress-induced endothelial DKK1; (3) shear stress-induced endothelial DKK1 promoted lipid uptake by co-cultured SMCs via upregulation of SR-A; (4) endothelial-specific knockout of DKK1 ameliorated atherosclerosis and lipid accumulation in SMCs within plaques; (5) endothelial-specific overexpression of DKK1 exacerbated AS and promoted SMC-derived foam cell formation *in vivo*; and (6) DKK1-inhibited proteasomal degradation of SR-A via USP53-dependent K48 polyubiquitination to promote foam cell formation in SMCs. This study focused on the paracrine effect of endothelial-secreted DKK1 induced by low shear stress on SMC-derived foam cell formation and the underlying mechanisms.

Vascular ECs sense hemodynamic changes directly and activate mechanical signal transduction pathways to induce a series of intracellular reactions, resulting in EC dysfunction 36. Activated ECs secrete vasoactive factors that regulate the functions of adjacent SMCs 37-39. Laminar shear stress on ECs can promote the differentiation of co-cultured SMCs, thereby maintaining a contractile phenotype and inhibiting migration and proliferation 25. In contrast, when ECs are exposed to pathological (low or oscillatory) shear stress, the expression of genes associated with a contractile phenotypic (e.g., ACTA2) was downregulated, and the expression of inflammatory factors, such as VCAM1, IL-8, and MCP-1, was increased in co-cultured SMCs 33. Furthermore, emerging evidence has substantiated the effect of ECs on cholesterol metabolism in co-cultured SMCs. ECs can reduce cholesterol levels by inhibiting the expression of the cholesterol biosynthesis gene, DHCR24, in SMCs in a form of spherical vascular organoids, thereby maintaining the contractile phenotype of SMCs 15. Another study found that exosomes derived from bovine aortic endothelial cells containing anti-miR-33a-5p promoted cholesterol efflux from bovine aortic SMCs 16. However, the mechanism by which ECs—as cells directly exposed to pathological shear stress at atherogenic susceptible sites—modulate the lipid metabolism of adjacent SMCs under LowSS remains unknown and requires further investigation. ECs exposed to pathological shear stress induce loss of the contractile phenotype in co-cultured SMCs 39, thereby enhancing the ability of SMCs to take up lipoproteins in the pathological environment of AS.

Previous studies have shown that endothelial DKK1 is upregulated under pathological shear stress and is essential for maintaining vascular homeostasis and promoting atherogenesis 21, 23. While the autocrine action of DKK1 as a pro-atherogenic factor for ECs has been established, the paracrine role of DKK1 in adjacent SMCs remains unclear. Abnormal lipid metabolism and foam cell formation are important pathological features of AS 40, 41. The pro-atherogenic role of DKK1 has been validated by numerous studies; however, its effects on plaque lipid metabolism and foam cell formation remain unclear. Previously, we demonstrated the inhibitory effect of DKK1 deficiency on lipid deposition in APOE^-/-^ mice 23. Simultaneously, DKK1 was identified as a target of lipid-lowering drugs of statins. Atorvastatin was found to decrease DKK1 expression in human umbilical vein endothelial cells and reduce DKK1 plasma levels in rabbits fed HFD. It is anticipated that DKK1 knockout can mimic the lipid-lowering effects of statins 42. However, despite the observation that DKK1 promotes plaque lipid deposition *in vivo*, previous studies have reported that DKK1 does not induce an increase in lipid accumulation within macrophages, and that knockdown of DKK1 does not inhibit the formation of foam cells derived from macrophages 24, 43, 44. SMCs are an important source of foam cell populations within plaques, and studies have confirmed that at least 50% of foam cells in the human coronary artery intima and 70% in APOE^-/-^ mice originate from SMCs rather than from macrophages 12, 13. The role of foam cells derived from SMCs within plaques is increasingly being recognized. However, the effects of DKK1 on SMC-derived foam cell formation remain unclear. The objective of this study was to investigate the paracrine effect of DKK1 secreted by ECs at AS-susceptible sites that produce pathological shear stress on lipid deposition in adjacent SMCs. We demonstrated that DKK1 serves as a paracrine mediator secreted by ECs to act on co-cultured SMCs under LowSS conditions, promoting lipid accumulation by upregulating the expression of SR-A in SMCs. *In vivo*, the endothelium-specific knockout of DKK1 (DKK1^ECKO^/APOE^-/-^) ameliorated, while endothelium-specific overexpression of DKK1 (DKK1^ECTg^) exacerbated lipid deposition and SMC-derived foam cell formation in AS. These results suggest that endothelial DKK1 acts as a key intercellular mediator that regulates foam cell formation in SMCs, and that its release is promoted by atherogenic LowSS.

Acquisition of the CD68 marker and loss of the ACTA2 marker in SMC-derived foam cells hampers their identification within plaques. Recently, several studies have documented the use of CD45 as a leukocyte-specific marker that is not expressed by SMCs 45. CD45 has been used to distinguish leukocytes from non-leukocytes within plaques based upon their positivity and negativity, respectively. This has been used to compare differences in autophagy flux and LAL expression levels between the two populations 46, 47. In this study, the CD45 marker and BODIPY493/503 were used to differentiate between foam cells populations derived from leukocytes and non-leukocytes within atherosclerotic plaques. We identified SMC-derived foam cells based on CD45 negativity and BODIPY493/503 positivity. ACTA2, MYH11, TAGLN, and PDGFRβ were also used to label SMCs in plaques, whereby ACTA2, MYH11, and TAGLN are specific markers for the contractile phenotype of SMCs. PDGFRβ was used are a marker of the synthetic phenotype of SMCs 48. SMC lineage tracing is the most direct approach to investigate foam cell formation in SMCs, and should be used to validate our findings in future studies. SMC-derived foam cells lose their contractile phenotype, transition to the proliferative type, release inflammatory factors, release elastase, transition to a macrophage-like state, downregulate ABCA1 expression, and lose their matrix arrangement ability 49, 50. These results suggest that SMCs lose their original function after foaming and maybe involved in proinflammatory processes, aggravating lipid deposition and promoting plaque instability and AS progression. This suggests that abnormal lipid metabolism by SMCs to form foam cells may accelerate plaque progression, and that inhibiting foam cell formation in SMCs may represent a therapeutic target for AS.

Our understanding of foam cells originates from research on macrophages, which has contributed to studies of SMC-derived foam cell formation. Among the lipid uptake receptors, scavenger receptors are central to the recognition and uptake of modified LDL 51, 52. SR-A and CD36 account for 75-90% of the total internalized oxLDL in macrophages 53, and inhibition of SR-A and CD36 expression in macrophages significantly reduces foam cell formation 54, 55. However, few studies have focused on the role of SR-A and CD36 in the formation of SMC-derived foam cells. In our study, interfering with DKK1 expression in ECs, and using a DKK1-neutralizing antibody significantly alleviated the upregulation of LowSS in SR-A expression in SMCs in a co-culture model, with no effect on CD36 expression. These results suggest that DKK1 promotes lipid deposition by modulating SR-A expression in SMCs. Further experiments showed that DKK1 did not influence SR-A transcription, but regulated its protein expression through the UPP system. Transcriptome sequencing revealed that expression of USP53, a deubiquitinating enzyme, was the only gene that exhibited significant differences among UPP-related genes following interference with DKK1 expression or stimulation with exogenous rDKK1**.** Furthermore, Co-IP experiments confirmed endogenous and exogenous interactions between USP53 and SR-A. These results suggest that USP53 may represent an intermediate molecular mechanism that mediates DKK1 to regulate the expression of SR-A in SMCs and influence the formation of foam cells.

UPP is an ATP-dependent, reversible pathway that degrades up to 80% of intracellular proteins 56. UPP is involved in various biological processes, including transcription, the cell cycle, the immune response, and inflammatory reactions 57, 58. Clinical studies have revealed that UPP may play an important role in the development of AS 59, 60. Deubiquitinating enzymes are a large family of proteases, including USP53, that remove ubiquitin chains from target proteins, controlling their function and stability 56. Studies have shown that USP14, a member of the same family as USP53, inhibits the ubiquitination of macrophage CD36, stabilizes CD36 protein expression, and promotes macrophage-derived foam cell formation 61. However, the role of USP53 in foam cell formation and AS remains unclear. In this study, for the first time, we showed that the expression of USP53 in SMCs was gradually upregulated with continued HFD feeding. In addition, USP53 expression in severe plaques in humans was higher than that in mild plaques, indicating that USP53 may play an important role in AS.

Intriguingly, USP53 is devoid of deubiquitinating activity owing to the lack of an essential histidine residue in its catalytic pocket 62. However, recent studies have suggested that USP53 retains some catalytic activity as a deubiquitinating enzyme. USP53 enhanced the stability of cytochrome c (CYCS) in hepatocellular carcinoma (HCC) cells by blocking ubiquitination and subsequent degradation to induce apoptosis 63. Furthermore, USP53 can regulate the stability of β-catenin through its interaction with FBXO31 64. This is consistent with our finding that USP53 interacts with, and deubiquitinates SR-A, thereby stabilizing SR-A and promoting the formation of SMC-derived foam cells. There are many types of ubiquitination, with Lys48- and Lys63-linked polyubiquitination being the most common and well-characterized 56. Lys48-linked polyubiquitin usually leads to proteasomal degradation, whereas Lys63-linked chains are signatures of intracellular trafficking, signal transduction, and DNA repair 65, 66. Endogenous and exogenous ubiquitination, in addition to *in vitro* ubiquitination assays, have revealed that SR-A is deubiquitinated by USP53 through its deubiquitinating enzyme via K48-linked polyubiquitination, which inhibits protein degradation via the 26S proteasome pathway.

Thus, herein, we demonstrate that low shear stress-induced endothelial DKK1 promotes the formation of co-cultured SMC-derived foam cells, which is primarily mediated by the USP53-inhibited proteasomal degradation of SR-A. USP53 upregulates the SR-A expression via a deubiquitinating function, leading to stronger and more stable promotion of foam cell formation and AS. Our findings suggest that the DKK1/USP53 axis represents a potential novel therapeutic target for AS.

There are several limitations to this study. First, further studies should investigate the pathway of endothelial DKK1 uptake by SMCs. Second, the therapeutic efficacy of the DKK1 neutralizing antibody in murine AS remains to be determined.

## Conclusions

In conclusion, our study showed that low shear stress-induced EC-secreted DKK1 can be taken up by co-cultured SMCs to promote SMC-derived foam cell formation. Specific deficiency or overexpression of DKK1 in ECs inhibits or promotes atherosclerotic plaques and SMC-derived foam cell formation, respectively, *in vivo*. Mechanistically, DKK1 regulates SR-A protein stability via USP53-dependent proteasomal degradation. USP53 interacts with SR-A and regulates K48-linked polyubiquitination in SMCs, which may be enhanced by endothelial DKK1. Our study reveals a broad effect of endothelial DKK1 expression. Targeting endothelial DKK1 can inhibit the detrimental effects on ECs in an autocrine manner, and partially alleviate the deleterious effects on adjacent SMCs in a paracrine fashion. As a novel target for cancer therapy, our study supports a role for DKK1 in treating AS further; thus, this may achieve the therapeutic purpose of "killing two birds with one stone" by simultaneously targeting tumors and inhibiting vascular lesions.

## Supplementary Material

Supplementary methods, figures and tables.

## Figures and Tables

**Figure 9:**
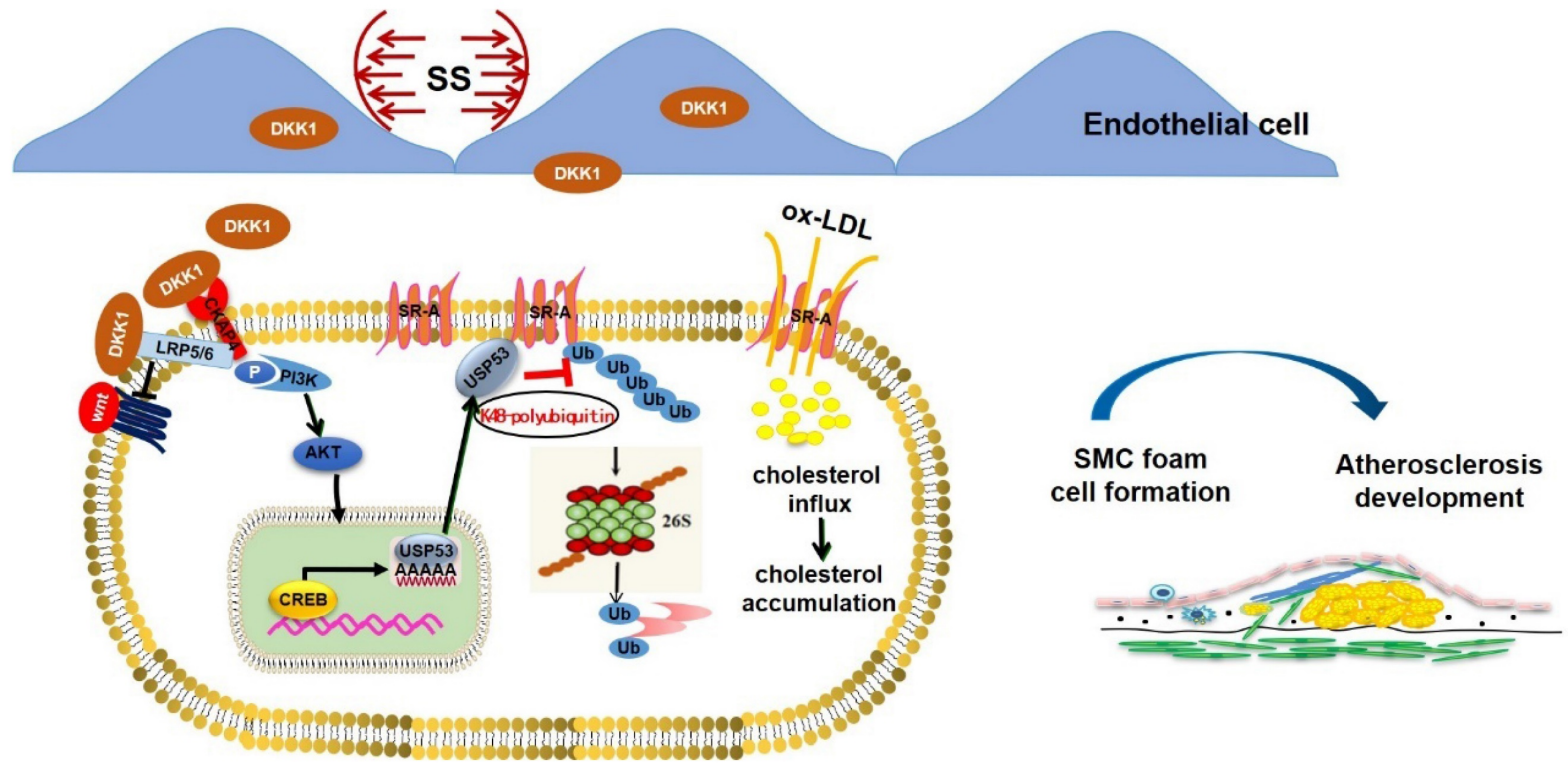
** Graphical abstract.** Endothelial DKK1 induced by pathological low shear stress is a key intercellular mediator that promotes foam cell formation of adjacent SMCs by upregulating SR-A via deubiquitinated by USP53.

## Abbreviations

AA: Aortic arch
AAV: Adeno-associated virus
ACTA2: α-smooth muscle actin
AS: Atherosclerosis
CKAP4: Cytoskeleton associated protein 4
DHCR24: 24-dehydrocholesterol reductase
DKK1: Dickkopf-1
ECs: Endothelial cells
FBXO31: F-box only protein 31
HAEC: Human aortic endothelial cells
HASMC: Human aortic smooth muscle cells
HDL-c: High density lipoprotein-cholesterol
HFD: High-fat diet
IL-8: Interleukin-8
LAL: Lysosomal Acid Lipase
LCA: Left common carotid artery
LDL-c: Low-density lipoprotein cholesterol
LowSS: Low shear stress
LRP6: Low density lipoprotein receptor related protein 6
MCP-1: Monocyte chemoattractant protein 1
MYH11: Myosin heavy chain 11
RCA: Right common carotid artery
SMCs: Smooth muscle cells
SR-A: Scavenger receptor-A
TA: Thoracic aorta
TAGLN: Transgelin
TC: Total cholesterol
TG: Triglycerides
PCSK9: Proprotein convertase subtilisin/kexin type 9
PDGFRβ: Platelet-derived growth factor receptor β
USP53: Ubiquitin-specific protease 53
VCAM1: Vascular cell adhesion molecule 1
WT: Wild type
