# Supplementary material for: Endothelial Dickkopf-1 Promotes Smooth Muscle Cell-derived Foam Cell Formation via USP53-mediated Deubiquitination of SR-A During Atherosclerosis
Source: Int J Biol Sci. 2024 May 19;20(8):2943–64. doi: 10.7150/ijbs.91957 (PMC11186357; doi:10.7150/ijbs.91957)
Supplement: Supplementary file 1 — Supplementary methods, figures and tables. [file ijbsv20p2943s1.pdf]

Supplementary Material

Endothelial Dickkopf-1 Promotes Smooth Muscle Cell-derived Foam Cell Formation via USP53-mediated Deubiquitination of SR-A During Atherosclerosis

Xiaolin Liu[1], Tengfei Zheng[1], Yu Zhang[1], Yachao Zhao[1], Fengming Liu[2], Shen Dai[3], Meng Zhang[1], Wencheng Zhang[1], Cheng Zhang[1], Mei Zhang[1,*], Xiao Li[1,*]

1. National Key Laboratory for Innovation and Transformation of Luobing Theory; The Key Laboratory of Cardiovascular Remodeling and Function Research, Chinese Ministry of Education, Chinese National Health Commission and Chinese Academy of Medical Sciences; Department of Cardiology, Qilu Hospital of Shandong University, Jinan, China.

2. Department of Immunology, School of Basic Medical Sciences, Shandong University, Jinan, China.

3. Department of Physiology & Pathophysiology, School of Basic Medical Sciences, Shandong University, Jinan, China.

# These authors contributed equally to this work.

[*] **Corresponding author:**

Mei Zhang, Department of Cardiology, Qilu Hospital of Shandong University, No. 107, Wen Hua Xi Road, Jinan, Shandong, 250012, China.

E-mail: zhangmei@email.sdu.edu.cn

Xiao Li, Department of Cardiology, Qilu Hospital of Shandong University, No. 107, Wen Hua Xi Road, Jinan, Shandong, 250012, China.

E-mail: lixiao560718@163.com

Supplementary methods

Partial carotid ligation surgery

All animal experiments were conducted following the National Institutes of Health Guide for the Care and Use of Laboratory Animals and approved by the Medical Ethics Committee of Shandong University, Jinan, China (DWLL-2019-097). Partial carotid ligation model was used to induce low shear stress [1]. Male mice (C57BL/6J background) provided by Vital River Laboratory Animal Technology were reared at 8-10 weeks of age and preformed partial left carotid ligation surgery. In brief, anesthesia was induced by mini-pump placement with oxygen and 1-2% isoflurane and a depilatory cream was applied to the depilated area. A 4-6 mm vertical incision was made in the middle of the neck. Left common carotid artery (LCA) was separated to expose four distal branches of the external carotid artery, internal carotid artery, occipital artery and superior thyroid artery. Three of the four branches (external carotid, internal carotid, and occipital arteries) were ligated, leaving the superior thyroid artery intact. The incision is then sutured. During recovery, mice were placed in a warm room to restore body temperature. Each mouse used its right carotid artery as control for the sham operation. After the surgery, mice were fed with HFD consisting of 40% fat and 1.25% cholesterol (TROPHIC Animal Feed High-Tech Co. Ltd, China, TP28521) for 4 weeks to establish atherosclerosis model. Mice were weighed before euthanized by intravenous injection of a lethal dose of pentobarbital sodium (200 mg/kg) and then the apical blood, whole aortas from the aortic root to the iliac bifurcation and left common carotid arteries were obtained. All operations and subsequent analysis were performed in a blinded fashion.

En face analysis

Configure fresh Oil Red O reserve solution firstly: add 0.5 g Oil Red O powder (1320-06-5, Sigma-Aldrich, St. Louis, MO, USA) into 100 ml isopropyl alcohol solution followed by stirring at 60°C for 2 h until fully dissolved, filtering once using 0.22 μm filters and store at 4°C. Configure fresh Oil Red O working solution: mix

reserve solution with ddH_2O (3:2) followed by filtering twice using 0.22 μm filters before use. The aorta was cleaned under the general microscope and opened longitudinally. The aorta was stained by Oil red O working solution for 2 h in the room temperature, immersed in hot water, and pressed to flat followed by photographed with a digital camera (Nikon, Japan). The total surface area and Oil Red O-stained positive lesion area were evaluated using Image-Pro Plus 6.0 software. The total surface area occupied by the Oil Red O-positive area was assessed.

Histological analysis

Vascular tissues were soaked in 4% paraformaldehyde (P1110, Solarbio) for at least 24 h. Adventitial tissue of the whole aorta and carotid arteries were removed carefully under the general microscope. Then vessel tissues were flushed overnight and embedded by OCT (4583, SAKURA, Japan) in temperatures of at least -20°C. To analyze the morphology and composition of carotid plaque, frozen tissue serial slices (5 μm thick) were obtained. Digital image analysis system such as Image-Pro Plus 6.0 or Image J were used to analyze images from slices stained with hematoxylin-eosin (H&E), Oil Red O, immunohistochemistry or Masson staining, and the plaque lesion or positive staining areas were evaluated.

Hematoxylin-eosin (H&E) staining

Frozen tissue slices were balanced at room temperature at least 1 h. After 10 min of hydration using tap water, frozen sections were washed using 1×PBS solution. Then, frozen tissue slices were incubated for 2 min in hematoxylin stain (G1140， Solarbio), soaked in 1% hydrochloric acid alcohol (1 ml HCL dissolved in 100 ml 70% alcohol) for 5 s, and then rinsed with tap water for 6-7 min. The nuclei appear blue and cytoplasm is not stained under optical light microscope (Olympus, Japan). After that, slices were stained with 0.5% eosin (G1100, Solarbio) for 1 min and rinsed with the running tap water for 30 s. After being dehydrated with 75% ethanol for 2 min, 85% ethanol for 2 min, 95% ethanol for 5 min, 100% ethanol for 5 min and xylene for 5 min, slices are sealed with neutral gum (G8590, Solarbio). Histopathological images

were acquired with Pannoramic Scan II digital slide scanner (3DHistech, Budapest, Hungary). Lesion area (mm^2) was measured using Image J software.

Oil Red O staining

After hydration, plaque tissue slices were then soaked in fresh Oil Red O working solution for 10 min followed by soaked in 37°C hot ddH_2O for 30 s. Plaques were stained red under optical light microscope. Slices were sealed using glycerin gelatin seal tablet (S2150, Solarbio). Histopathological images were acquired with Pannoramic Scan II digital slide scanner (3DHistech, Budapest, Hungary). The total lumen area occupied by the Oil Red O-positive area was assessed using Image J software.

Immunohistochemistry staining

For immunohistochemistry staining, serial frozen tissue slices of carotid plaque were hydrated for 10 min, incubated with 3% H_2O_2 for 20 min and blocked with 5% bovine serum albumin (BSA) (A8020, Solarbio) at room temperature for 1 h. After that, slices were incubated with primary antibody against SR-A (1:100 dilutions, ab271070, abcam, UK) for SR-A detection or USP53 (1:100 dilutions, GTX87956, GeneTex, China) for USP53 detection overnight at 4°C. Horseradish peroxidase (HRP)-conjugated goat anti-rabbit IgG antibody (PV-9001, ZSGB-BIO, Beijing, China) was added on slices on the second day at room temperature for 1 h and DAB Substrate (3,3-diaminobenzidine) (ZLI-9018, ZSGB-BIO, Beijing, China) was added for indicated time to show positive area (brown). Staining with hematoxylin for 2 min and soaked in 1% hydrochloric acid alcohol for 5 s to stain nuclei followed by re-hydration following the above steps. After that, slices were sealed with neutral gum. Histopathological images were acquired with Pannoramic Scan II digital slide scanner (3DHistech, Budapest, Hungary). The total plaque area occupied by the SR-A or USP53 positive area was assessed using Image Plus Pro 6.0 software.

Immunofluorescence staining

For immunofluorescence staining, serial frozen tissue slices of carotid plaque or arteries were hydrated for 10 min, treated with 0.5% Triton-X 100 (ST79, Beyotime,

Shanghai, China) in PBS at room temperature for 20 min, blocked with 5% BSA at room temperature for 1 h and incubated overnight at 4°C with the following primary antibodies. For double immunofluorescence staining for DKK1 and CD31, tissue slices were incubated with both a mouse anti-DKK1 antibody (1:100 dilution, ab61275, abcam, UK) and a rabbit anti-CD31 antibody (1:100 dilutions, ab28364, abcam, UK). On the second day, the corresponding goat anti-rabbit Alexa Fluor plus 594 fluorescent secondary antibody (1:200 dilution, ab150080, abcam, UK) and goat anti-mouse Alexa Fluor plus 488 fluorescent secondary antibody (1:200 dilution, ab150113, abcam, UK) were added on slices for 1 h at room temperature. For double immunofluorescence staining for DKK1 and ACTA2, tissue slices were incubated with both a rabbit anti-DKK1 antibody (1:100 dilutions, ab109416, abcam, UK) and a mouse anti-ACTA2 antibody (1:200 dilutions, ab7817, abcam, UK). On the second day, the corresponding donkey anti-rabbit Alexa Fluor plus 488 fluorescent secondary antibody (1:200 dilution, ab150061, abcam, UK) and donkey anti-mouse Alexa Fluor plus 594 fluorescent secondary antibody (1:200 dilution, ab150112, abcam, UK) were added on slices for 1 h at room temperature. For double immunofluorescence staining for SR-A and ACTA2, tissue slices were incubated with both a rabbit anti-SR-A antibody (1:50 dilutions, ab271070, abcam, UK) and a mouse anti-ACTA2 antibody (1:200 dilutions, ab7817, abcam, UK) simultaneously. Corresponding donkey anti-rabbit Alexa Fluor plus 488 secondary antibody (1:200 dilution, ab150061, abcam, UK) and donkey anti-mouse Alexa Fluor plus 594 secondary antibody (1:200 dilution, ab150112, abcam, UK) were added on slices for 1 h at room temperature. For double immunofluorescence staining for USP53 and ACTA2, tissue slices were incubated with both a rabbit anti-USP53 antibody (1:50 dilutions, GTX87956, GeneTex, China) and a mouse anti-ACTA2 antibody (1:200 dilutions, ab7817, abcam, UK) simultaneously. Repeat the above procedure of fluorescent secondary antibody incubation. After washing three times with 1×PBS, slices were stained with undiluted Fluoroshield Mounting Medium with DAPI (ab104139, abcam, UK). Images were photographed using an electric upright microscope (Ni-E, DS-Ri2, Nikon, Japan).

Tissue BODIPY staining

Lipid droplets in carotid plaque were labeled with 10 μg/ml 4,4-difluoro-1,3,5,7,8-pentamethyl-4-bora-3a,4a-diaza-s-inda-cene (BODIPY) (D3922, Thermo Fisher Scientific) which was dissolved and diluted with DMSO (D8371, Solarbio) at room temperature for 30 min after the incubation with fluorescent secondary antibody. SMC -derived foam cells within mouse plaques were labeled by incubating with CD45 antibody (1:100 dilutions, ab33923, abcam, UK), ACTA2 antidody (1:200 dilutions, ab7817, abcam, UK), PDGFRβ antibody (1:50 dilutions, 3169T, Cell Signaling Technology, USA), MYH11 antibody (1:100 dilutions, YM6757, immunoway) or TAGLN antibody (1:100 dilutions, ab14106, abcam, UK) and BODIPY493/503. Images were obtained by a Zeiss LSM710 confocal microscope (Carl Zeiss, Oberkochen, Germany). Quantification was performed using Image J software after appropriate thresholding.

Serum lipid test

The apical blood was centrifuged at 3000 rpm for 15 min at 4°C to collect the upper serum. Serum concentration of total cholesterol (TC), triglycerides (TG), HDL-cholesterol (HDL-c) and LDL-cholesterol (LDL-c) were determined using Beckman automatic biochemical analyzer (Beckman Counter, AU5821) according to the manufacturer's Instructions.

Primary mouse lung Endothelial cells isolation and culture

Mouse lung endothelial cells were cultured as described [2, 3]. Briefly, 3-week-old mice were anesthetized and lung tissue were minced under sterile conditions and then digested with 1 mg/ml collagenase I (SCR103, Sigma) plus 60 units/ml DNase I (9003-98-9, Sigma) in PBS, and then heated at 37°C incubator for 45 min. Single cell suspension was filtered through a 70 μm cell filter and centrifuged. The cell pellet was resuspended in 0.1% BSA (diluted in PBS) containing anti-CD31 antibody conjugated Dynabeads (11155D, Invitrogen, Carlsbad, CA) added for 20 min with rotation. Dissociated on a magnetic separator, cells were plated on gelatin-coated T25 culture flasks. Endothelial cells were cultured in ECM containing 10% FBS (10099141,

Gibco, Grand Island, NY, USA) and 30 g/ml Endothelial Cell Growth Factor. When cells were 80% to 90% conjugative, they were sorted a second time with ICAM-2 antibody-conjugated Dynabeads. Cellular immunofluorescence staining for CD31 was used to identify endothelial cells. The cells were digested with trypsin (CN0004, Sparkjade Biotechnology Co., Ltd, Shandong, China).

Primary mouse aortic Endothelial Cells isolation and culture

Mouse aortic endothelial cells were extracted as described [4, 5]. Briefly, (Aseptic operation in ultra-clean table) 3-week-old mice were extracted the heart, sections from the aortic arch to the thoracic aorta (above the 12th rib) was separated and placed in cold PBS. Under the microscope, the peritubular adipose tissue and connective tissue were carefully removed and the aorta was dissected longitudinally with scalpel to obtain a 0.5-0.8 mm wide vascular band. Cold Matrigel Matrix (356231, Corning) was laid on the 24-well plate in advance (diluted to 200 μg/ml and operated on ice to prevent early solidification). The aortic band of each mouse was cut into small pieces about 1 cm long and the lumen was inoculated into the 24-well plate with Matrigel Matrix downwards (2~3 aortas were put into one well). 24-well plates were placed immediately and carefully in a cell incubator until the Matrigel Matrix freeze (at least 2 h). At least 2 ml ECM was added to each well and cultured for at least one week. When a mass of cells is seen crawling out (at a density of at least 80%, the time may be extended to 2 weeks. During this period, the medium was replaced every 3 days and careful not to touch the aorta tissue), the aorta was removed from the well and wash cells with sterile 1×PBS carefully for three times. Collecting cells with trypsin containing EDTA (25200056, Gibco) digestion and ECM containing 10% FBS termination of digestion followed by centrifugal. Cells were cultured in gelatin-coated flasks with ECM containing 10% FBS. Cell sorting with MACS antibody-conjugated Dynabeads: endothelial cells can also be purified by anti-CD31 antibody conjugated Dynabeads sorting after cell collection. (Same as magnetic bead sorting of lung endothelial cells). Cellular immunofluorescence staining for CD31 was used to identify endothelial cells.

Primary mouse aortic Vascular Smooth Muscle Cells (SMC) isolation and culture

Mouse aortic SMC were extracted as described [4]. In brief, the aorta of the mouse was separated to the iliac bifurcation, and the middle layer of the aorta was cut under the microscope, cutting into 4 mm long blood vessel segments and placed in a culture flask for culture. When a mass of cells is seen crawling out, cells can be digested and collected.

Immunofluorescence staining of cells

For immunofluorescent staining of cells, cells were cultured on circular coverslips of 24-well plates. Cells were fixed with 4% paraformaldehyde (P1110, Solarbio) at room temperature for 20 min followed by washed with 1×PBS. After that, cells were treated with 0.5% Triton-X 100 at room temperature for 20 min and blocked with 5% BSA at room temperature for 1 h. For mouse lung ECs or aortic ECs identification, cells were incubated with a rabbit anti-CD31 primary antibody (ab28364, abcam, UK) overnight at 4℃. For mouse aortic SMC identification, cells were incubated with a rabbit anti-ACTA2 primary antibody (ab5694, abcam, UK) overnight at 4℃. In the second day, cells were incubated with donkey anti-rabbit Alexa Fluor plus 488 secondary antibody at room temperature for 1 h. For colocalization staining of USP53 and SR-A in HASMCs, cells were incubated with a Mouse anti-USP53 primary antibody (H00054532-B01P, Abnova, USA) and a Rabbit anti-SR-A primary antibody (ab271070, abcam, UK) overnight at 4℃. CoraLite488-conjugated Goat Anti-Mouse IgG (H+L) (SA00013-1, Proteintech, USA) and Goat Anti-Rabbit IgG H&L (Alexa Fluor® 594) (ab150084, abcam, UK) were used for 1 h at room temperature. Cell coverslips were sealed with undiluted Fluoroshield Mounting Medium with DAPI and images were photographed using an electric upright microscope (Ni-E, DS-Ri2, Nikon, Japan).

Co-culture equipment of HAECs and HASMCs

As has been reported [6, 7], the co-culture equipment was established by culture of HAECs and HASMCs at the bottom and the top of a parallel-plate co-culture flow

system (hereinafter referred to as the insert) (353090, BD falcon, USA) respectively separated by a 0.4 μm PET membrane (Figure 1F). Both of the two sides were coated with 1% gelatin in advance. HAECs were first seeded onto the bottom side of the insert for 4-6 h. And then, the insert with endothelium side down was inserted into a 6-well plate containing endothelium culture medium and HASMCs were seeded onto the top side. The next day, the insert was placed on a blood flow shear stress instrument (STR-4000, Flexcell, USA) to stimulate HAECs with shear stress. Shanghai naturethink company provided parallel-plate co-culture flow system.

ELISA

DKK1 level in the cellular supernatant of co-cultured ECs or SMCs were measured using Human DKK1 ELISA kit (P282941, R&D, USA). Cellular supernatant was collected and centrifuged at 3000~5000 rpm for 10 min to aspirate the supernatant for testing.

Cellular Oil Red O staining

Cells were fixed with 4% paraformaldehyde at room temperature for 20 min and then washed with pre-cold 1×PBS for three times. Afterwards, HASMCs were stained with fresh Oil Red O working solution at room temperature for 1 h and washed with warm water for several times. After that, hematoxylin was used to stain the nucleus for 2 min and 1% hydrochloric Acid alcohol was use for differentiation for 10 s. Cell coverslips were soaked in PBS for 10 min and sealed using glycerin gelatin seal tablet. HASMCs co-cultured with HAECs were digested by trypsin and then collected and seeded on cell coverslips of 24-well plates for Oil Red O staining (Repeated the above experimental steps). Images were captured using 100× manual upright microscopy (Ni-U, DS-Ri2, Nikon, Japan) and analyzed by Image J software.

Cellular BODIPY staining

For cellular BODIPY staining, HASMCs were cultured on coverslips of 24-well plates and stimulated with 100 μg/ml oxLDL for 24 h after corresponding treatment. Cells were fixed with 4% paraformaldehyde at room temperature for 20 min and then washed with pre-cold 1×PBS for three times. 0.5% Triton X-100 was used to

permeate cytoplasmic membrane for 20 min and 5% BSA was used to block for 1 h. Lipid droplets in HASMCs-derived foam cells were stained by incubating with BODIPY at 10~15 μg/ml for 30 min at a cell incubator under dark environment followed by washing cells with 1×PBS for three times. Undiluted Fluoroshield Mounting Medium with DAPI was used to stain the nucleus. Images were photographed using an electric upright microscope (Ni-E, DS-Ri2, Nikon, Japan) and analyzed with Image J software.

Cellular DIL-oxLDL staining

For cellular DIL-oxLDL staining to show the effect of lipid uptake of HASMCs, HASMCs were cultured on cell coverslips and incubated with 20 μg/ml Dioctadecyl-3,3,3,3-tetramethylin docarbocyanine-oxLDL (DIL-oxLDL, YB-0010, Yiyuan Biotech, Guangdong, China) at a 37°C incubator for 4 h after transfection. Afterwards, cells were fixed with 4% paraformaldehyde at room temperature for 20 min and then washed with pre-cold 1×PBS for three times. Finally, cell coverslips were sealed with undiluted Fluoroshield Mounting Medium with DAPI and images were photographed using an electric upright microscope (Ni-E, DS-Ri2, Nikon, Japan). Image J software was used to analyze the red fluorescent particles area per cell.

Adenoviruses construction and infection

Adenovirus containing plasmids expressing USP53 (Ad-USP53, Gene ID: 54532) was constructed by Weizhen Biotechnology (Shanghai, China) Co., Ltd. For overexpression of USP53, HASMCs (5×10^5 cells/well) were cultured in 6-well plates. Transfecting HASMCs for 12 h. At this time, cell supernatant was discarded and replaced with fresh SMCM for another 48 h. Fluorescent images were obtained using a manual inverted microscope (Ti-S, DS-Ri2, Nikon, Japan).

Plasmid construction and infection

Flag-tagged USP53 (Flag-USP53), Myc-tagged SR-A (Myc-SR-A), HA-tagged Ubiquitin (HA-Ub), HA-tagged K48, HA-tagged K63 and Flag-tagged USP53-C41S mutant plasmids were constructed by Biosune Biotechnology (Shanghai, China) Co.,

Ltd. To obtain Flag-USP53-C41S mutant plasmid, we performed a point mutation on the Cys-box of USP53 (33-50LNEPGQNSCFLNSAVQVL), which is indispensable for its catalytic properties. Tagged plasmids were transfected into HEK293T with Lipofectamine 3000 and Lipofectamine P3000 (L3000015, Invitrogen, USA) according to the manufacturer's instruction.

Western blot analysis

Cells and tissues were lysed with high efficiency RIPA lysis buffer (R0010, Solarbio, Beijing, China) of protease inhibitor PMSF (P0100, Solarbio, Beijing, China) was added. Phosphatase inhibitor (CW2383S, CWBiotech, China) was used to detect the expression of phosphorylated protein molecules. Protein lysates of mouse whole aorta were extracted using protein extraction kit (SD-001/SN-002, Invent, USA). Lung and kidney tissues of mouse were cut up with scissors for 15 min and lysed on ice in RIPA+PMSF lysis buffer for 15 min. BCA kit (23227, ThermoFisher Scientific) was used to detect protein concentration. The equal quantities of protein extracts were separated by 10% SDS-PAGE gel and then transferred to the PVDF membrane (ISEQ00010, Millipore, Billerica, MA, China). 5% milk was used to block nonspecific binding sites on Western bands at room temperature for at least 1 h. Western Blot bands were incubated with indicated antibodies at 4°C overnight (at least 12 h). In the second day, Western Blot bands were washed three times for 10 min each time using 1×TBST. After incubation with secondary horseradish peroxidase (HRP)-conjugated antibodies for 1 h at room temperature, protein signals were detected using a luminescent image analyzer (Amersham Imager 680, GE, USA). Image J software was used to calculate the gray value for each lane of Western Blot bands. β-actin was used as the equal loading control. All antibodies used in this study are listed in Table S5.

RNA extraction and Quantitative real-time PCR (qPCR)

Total RNA was extracted using TRIzol reagent (10296010, Invitrogen, CA, USA). The concentration of total RNA was detected using a Nanodrop 2000 (Thermo Fisher Scientific, Madison, WI, USA) equipment. 1000 ng total RNA was reverse

transcribed using HiScript® III RT SuperMix (R323-01, Vazyme Biotech Co. Ltd, Nanjing, China). Quantitative real-time PCR was performed using ChamQ Universal SYBR qPCR Master Mix (Q711-03, Vazyme Biotech Co. Ltd, Nanjing, China). The data were normalized by GAPDH. The primer sequence for RT-PCR used in this study was constructed by Biosune Biotechnology Co., Ltd (Shanghai, China) provided in Table S4.

Coimmunoprecipitation (Co-IP) assays

HASMCs treated with or without rDKK1 were used to detect the endogenous interaction between USP53 and SR-A and rDKK1 was used to verify that DKK1 can promote the endogenous combination of the two proteins. HEK293T cells were transfected Flag-USP53 and Myc-SR-A simultaneously to detect the exogenous interaction between USP53 and SR-A. HEK293T cells were transfected with increasing amounts of Flag-USP53, 2 μg Myc-SR-A and 2 μg HA-Ub to detect the gradient degradation of SR-A by USP53. HASMCs transfected with siCtrl or siUSP53 were co-cultured with Lenti-GFP ECs or Lenti-GFP-DKK1 EC to investigate whether endothelial DKK1 mediates the degradation of SR-A by USP53. To detect the effect of USP53 binding SR-A on its ubiquitination level, siUSP53 was transfected into HASMCs followed by ubiquitin molecular plus K63-linkage specific polyubiquitin were detected. To verify the effect of USP53 binding SR-A on its ubiquitination level further, HEK293T cells were transfected into Flag-USP53, Myc-SR-A and HA-Ub simultaneously to detect the change of Ub. The presence of Flag-mutant-USP53 eliminated the deubiquitinating activity of USP53 towards SR-A. Coimmunoprecipitation (Co-IP) was performed using Immunoprecipitation Kits (PK10007, Proteintech, USA). In brief, cells were seeded on 10 cm dishes and lysed with NP-40 (P0013F, Beyotime, China) adding into PMSF by a ratio of 99 to 1. Cells were collected and centrifuged with 14000 rpm for 15 min at 4℃. Drain the upper supernatant and set aside on ice to use. Pretreatment of magnetic beads (B23201, Bimake, China): After the magnetic beads were shaken for 1 min, 60 μl beads were placed in a 1.5 ml EP tube and 300 μl of RIPA was added for washing, and magnetic

separation was carried out with the help of a magnetic rack. After discarding the supernatant, RIPA was added for repeated washing for another two times, and 300 μl of RIPA was added to resuspend the magnetic beads for use. RIPA diluted IP antibody or IgG antibody which were listed in Table S5 to a concentration of 20 μg/ mL and mixed with magnetic beads. Beads-Antibody complex solution was mixed on a turnover mixer for 15 min and were separated magnetically to discard the upper solution. 300 μl RIPA was added into pellets to suspend Beads-Antibody complex and were separated magnetically followed by adding into antigen protein, which were mixed on a turnover mixer overnight at 4℃. In the second day, Beads-Antibody-Antigen complex was separated magnetically and the upper solution was discarded. Pellets were suspended with 40 μl 1×SDS-PAGE Loading Buffer (CW0027S, CWBiotech, China). Western Blot was used to detect the IP protein.

Chromatin immunoprecipitation (ChIP)

ChIP assay to verify the binding of CREB to the USP53 promoter with or without rhDKK1 stimulation. SimpleChIPTM Enzymatic Chromatin IP kit (Magnetic Beads) (9003, Cell Signaling Technology) was used according to manufacturer’s instructions. The HASMCs were crosslinked with a 37% aqueous formaldehyde solution for 10 minutes, followed by the addition of 125 mM glycine for 5 minutes. Subsequently, the cells were collected in centrifuge tubes, sonicated, and subjected to immunoprecipitation using normal IgG (ab172730, abcam, UK) or CREB (9197S, Cell Signaling Technology) antibodies at 4°C overnight. After elution and reverse crosslinking, the antibody/DNA complexes and DNA were purified using DNA purification methods and analyzed in duplicate by PCR with primer pairs targeting a specific region of the USP53 promoter. Primers for putative USP53 binding sites with CREB were: 5′-CTGAAACCCACAGAACCCCG-3′ forward and 5′-GGATTTCCACGGCAGACGC-3′ reverse.

Transcriptome sequencing

Transcriptome sequencing was completed by Shanghai Origingene Bio-pharm Technology Co. Ltd (Shanghai, China). Total RNA was extracted from HASMCs with

or without rDKK1 stimulation or siDKK1 transfection as described above. PCR products were purified using AMPure XP system and library quality was assessed on the Agilent Bioanalyzer 2100system. The library preparations were sequenced on a lllumina Novaseq6000 platform and paired-end reads were generated, and bioinformatics analysis. Briefly, the routine experimental procedures of Transcriptome Sequencing include RNA extraction and quality control, transcriptome sequencing library construction, library construction and computer sequencing, bioassay process-data storage.

References

1. Nam D, Ni CW, Rezvan A, et al. Partial carotid ligation is a model of acutely induced disturbed flow, leading to rapid endothelial dysfunction and atherosclerosis. Am J Physiol Heart Circ Physiol. 2009; 297: H1535-43.
2. Zhuang T, Liu J, Chen X, et al. Cell-Specific Effects of GATA (GATA Zinc Finger Transcription Factor Family)-6 in Vascular Smooth Muscle and Endothelial Cells on Vascular Injury Neointimal Formation. Arterioscler Thromb Vasc Biol. 2019; 39: 888-901.
3. Ghatnekar A, Chrobak I, Reese C, et al. Endothelial GATA-6 deficiency promotes pulmonary arterial hypertension. Am J Pathol. 2013; 182: 2391-406.
4. Molina-Sánchez P, Andrés V. Isolation of Mouse Primary Aortic Endothelial Cells by Selection with Specific Antibodies. Methods Mol Biol. 2015; 1339: 111-7.
5. Wang JM, Chen AF, Zhang K. Isolation and Primary Culture of Mouse Aortic Endothelial Cells. J Vis Exp. 2016; (118): 52965.
6. Guo Q, Huang F, Qing Y, et al. Decreased Jagged1 expression in vascular smooth muscle cells delays endothelial regeneration in arteriovenous graft. Cardiovasc Res. 2020;116(13):2142-155.
7. Chiu JJ, Chen LJ, Lee PL, et al. Shear stress inhibits adhesion molecule expression in vascular endothelial cells induced by coculture with smooth muscle cells. Blood. 2003; 101: 2667-74.

Supplementary figures

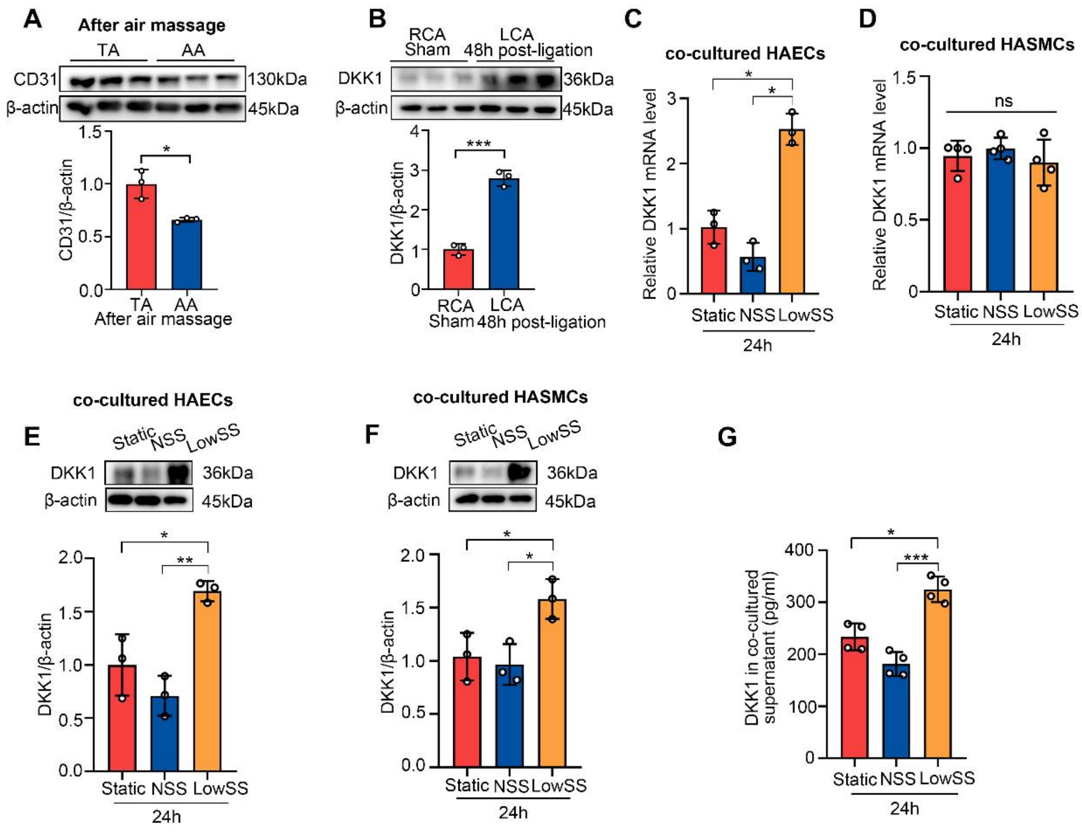

Figure S1 Low shear stress (LowSS) upregulated Dickkopf-1 (DKK1) expression in co-cultured HAECs compared with Static or Normal Shear Stress (NSS) group, which could be taken up by co-cultured HASMCs. (A) Western blot and quantification of CD31 in the AA and TA of C57BL/6J mice after air massage (n = 3). **(B)** Western blot (n = 3) of DKK1 in the LCA 48 h following partial ligation in C57BL/6J mice. The un-ligated RCA was used as a sham group. **(C)** RT-qPCR analysis for DKK1 mRNA level of co-cultured HAECs treated with Static, NSS and LowSS for 24 h (n = 3). **(D)** RT-qPCR analysis for DKK1 mRNA level of co-cultured HASMCs whose opposite ECs side were treated with Static, NSS and LowSS for 24 h

(n = 4). **(E, F)** Western blot of DKK1 in co-cultured HAECs (E, n = 3) and HASMCs (F, n = 3) in the indicated group. **(G)** Secretion level for DKK1 in the supernatant of co-cultured HAECs and HASMCs in the indicated group (n = 4). $^{*}p < 0.05$, $^{**}p < 0.01$, $^{***}p < 0.001$, ns not significant. Data are shown as the Mean ± standard deviation (SD). *p* values were calculated by One-way ANOVA followed by Tukey's multiple comparison test.

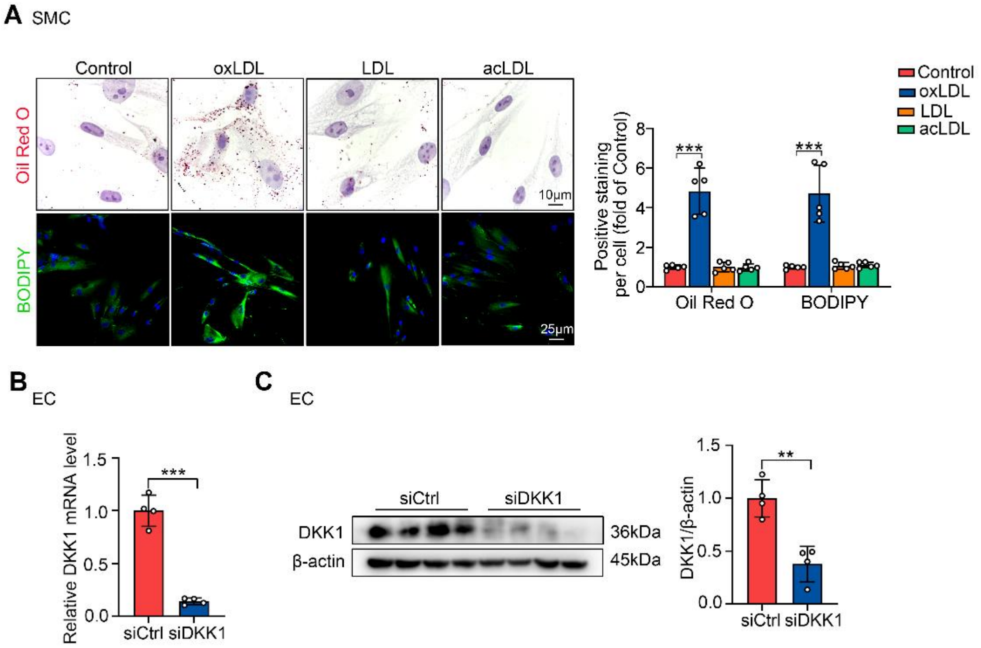

Figure S2 Establishment of SMC-derived foam cell model and DKK1 interference efficiency in HAECs. (A) Representative images and quantification of Oil Red O staining (upper panel) and BODIPY493/503 staining (lower panel) in SMCs incubated with oxLDL, LDL or acLDL (100 μg/ml) for 24 h (n = 5). **(B)** mRNA level of DKK1 in HAECs with or without siDKK1 transfection for 24 h (n = 4). **(C)** Representative Western blot image and quantitative analysis of DKK1 in HAECs with or without siDKK1 transfection for 48 h (n = 4). $^{**}p < 0.01$, $^{***}p < 0.001$. Data are shown as the Mean ± SD. Statistical analysis was performed with two-tailed unpaired Student's *t*-test.

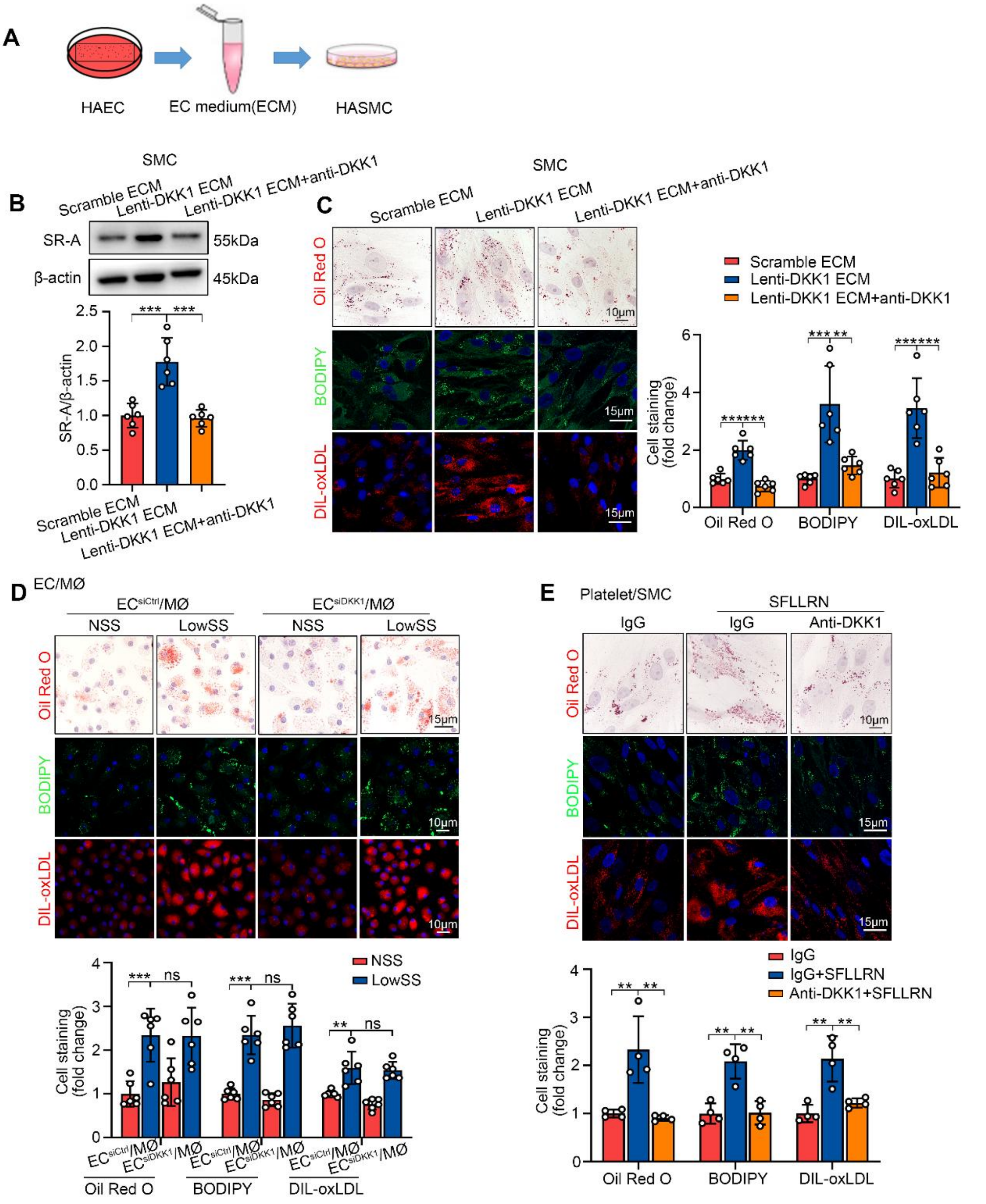

Figure S3

(A) Schematic illustration of the procedure how the HASMCs were incubated with ECM of HAECs transfected with lenti-DKK1. **(B)** Protein expression and

quantification of SR-A in SMCs incubated with DKK1 overexpressed-EC medium with or without DKK1 neutralizing antibody (n = 6). **(C)** Oil Red O (upper panel), BODIPY493/503 (middle panel), DIL-oxLDL (lower panel) staining and quantification SMCs incubated DKK1 overexpressed-EC medium with or without DKK1 neutralizing antibody (n = 6). **(D)** Oil Red O (upper panel), BODIPY493/503 (middle panel), DIL-oxLDL (lower panel) staining and quantification of macrophages co-cultured with ECs transfected with Control-siRNA (siCtrl) or DKK1 interference siRNA (siDKK1) under shear stress conditions. **(E)** Oil Red O (upper panel), BODIPY493/503 (middle panel), and DIL-oxLDL (lower panel) staining and quantification of co-cultured SMCs with platelets incubated with a DKK1-neutralizing antibody (10 μg/mL) or control IgG. $^{**}p < 0.01$, $^{***}p < 0.001$, ns not significant. Data are shown as the Mean ± SD. *p* values were calculated by Two-tailed unpaired Student's *t*-test or One-way ANOVA followed by Tukey's multiple comparison test.

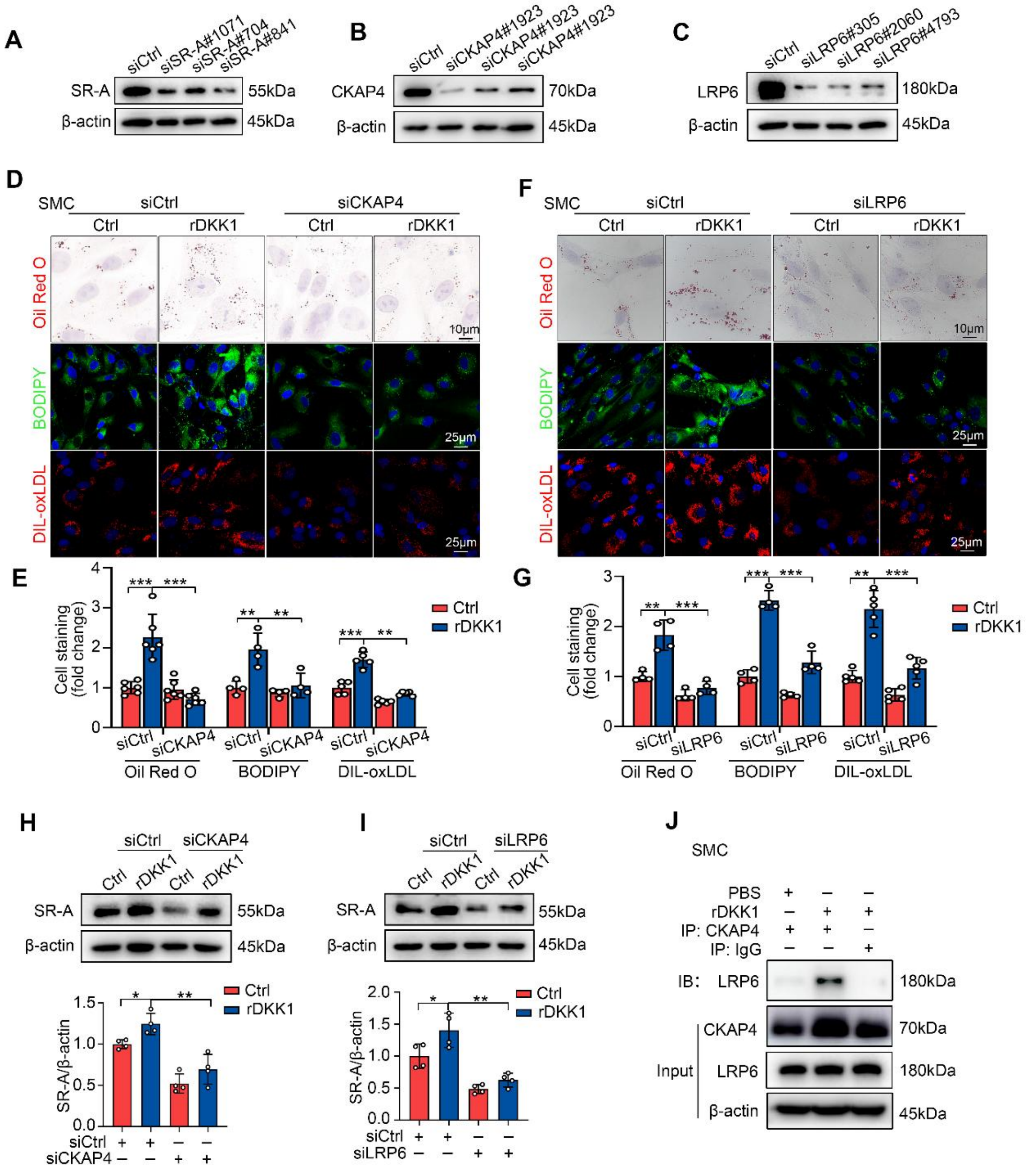

Figure S4 CKAP4 and LRP6 receptor mediated exogenous DKK1 to promote SMC-derived foam cell formation and SR-A expression. (A, B, C) Interference efficiency of SR-A (A), CKAP4 (B) and LRP6 (C) in HASMCs by siRNAs. **(D, E)** Oil Red O (upper panel, n = 6), BODIPY493/503 (middle panel, n = 4) and DIL-oxLDL (lower panel, n = 5) staining (D) and corresponding quantification (E) of SMCs transfected with siCtrl or siCKAP4 with or without rDKK1 incubation. **(F, G)** Oil Red O (upper panel, n = 4), BODIPY493/503 (middle panel, n = 4) and DIL-oxLDL (lower panel, n = 5) staining images (F) and corresponding quantification

(G) of SMCs transfected with siCtrl or siLRP6 with or without rDKK1 incubation. **(H)** Western blot for SR-A in SMCs transfected with siCtrl or siCKAP4 with or without rDKK1 incubation (n = 4). **(I)** Western blot for SR-A in SMCs transfected with siCtrl or siLRP6 with or without rDKK1 incubation (n = 4). **(J)** Co-IP assay investigating the endogenous interaction between USP53 and SR-A in HASMCs after rDKK1 incubation. $^{*}p < 0.05$, $^{**}p < 0.01$, $^{***}p < 0.001$. Data are shown as the Mean ± SD. Statistical analysis was performed with One-way ANOVA followed by Tukey's multiple comparison test.

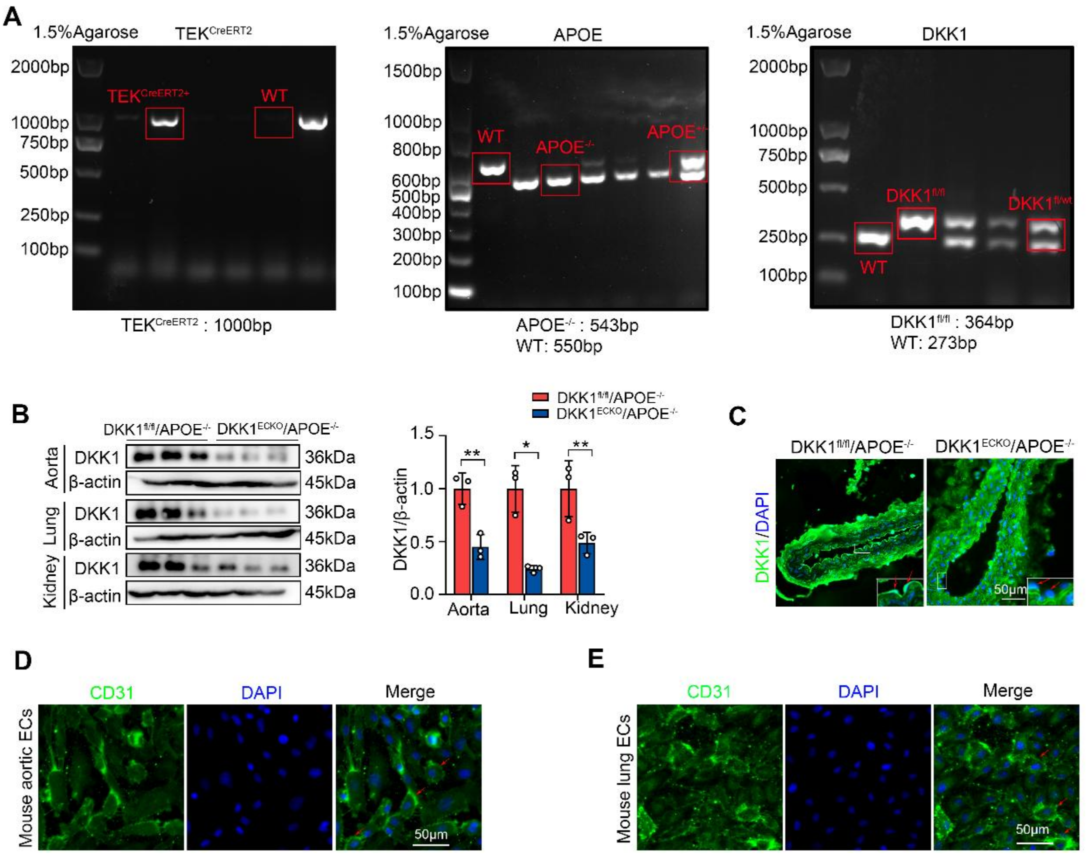

Figure S5 Identification of Endothelial Cells-specific DKK1 knockout in APOE$^{-/-}$ mice (DKK1^{ECKO}/APOE$^{-/-}$). (A) Genotyping DKK1 knockout mice by PCR and 1.5% agarose gel showed different genotypes as indicated in the label. **(B)** Representative Western blot images and quantitative analysis of DKK1 in aorta, lung and kidney tissues from DKK1$^{fl/fl}$/APOE$^{-/-}$ and DKK1^{ECKO}/APOE$^{-/-}$ mice (n = 3). **(C)** Representative immunofluorescence staining images for DKK1 (green) in carotid arteries from DKK1$^{fl/fl}$/APOE$^{-/-}$ and DKK1^{ECKO}/APOE$^{-/-}$ mice, which indicated the expression of DKK1 in endothelium (red arrows). **(D, E)** Primary mouse aortic ECs or primary mouse lung ECs were identified with immunofluorescence staining for CD31 (green). Red arrows indicate that CD31 is expressed on the surface of endothelial cell membrane. $^{*}p < 0.05$, $^{**}p < 0.01$. Data are shown as the Mean ± SD. Statistical analysis was carried out using two-tailed unpaired Student's *t*-test.

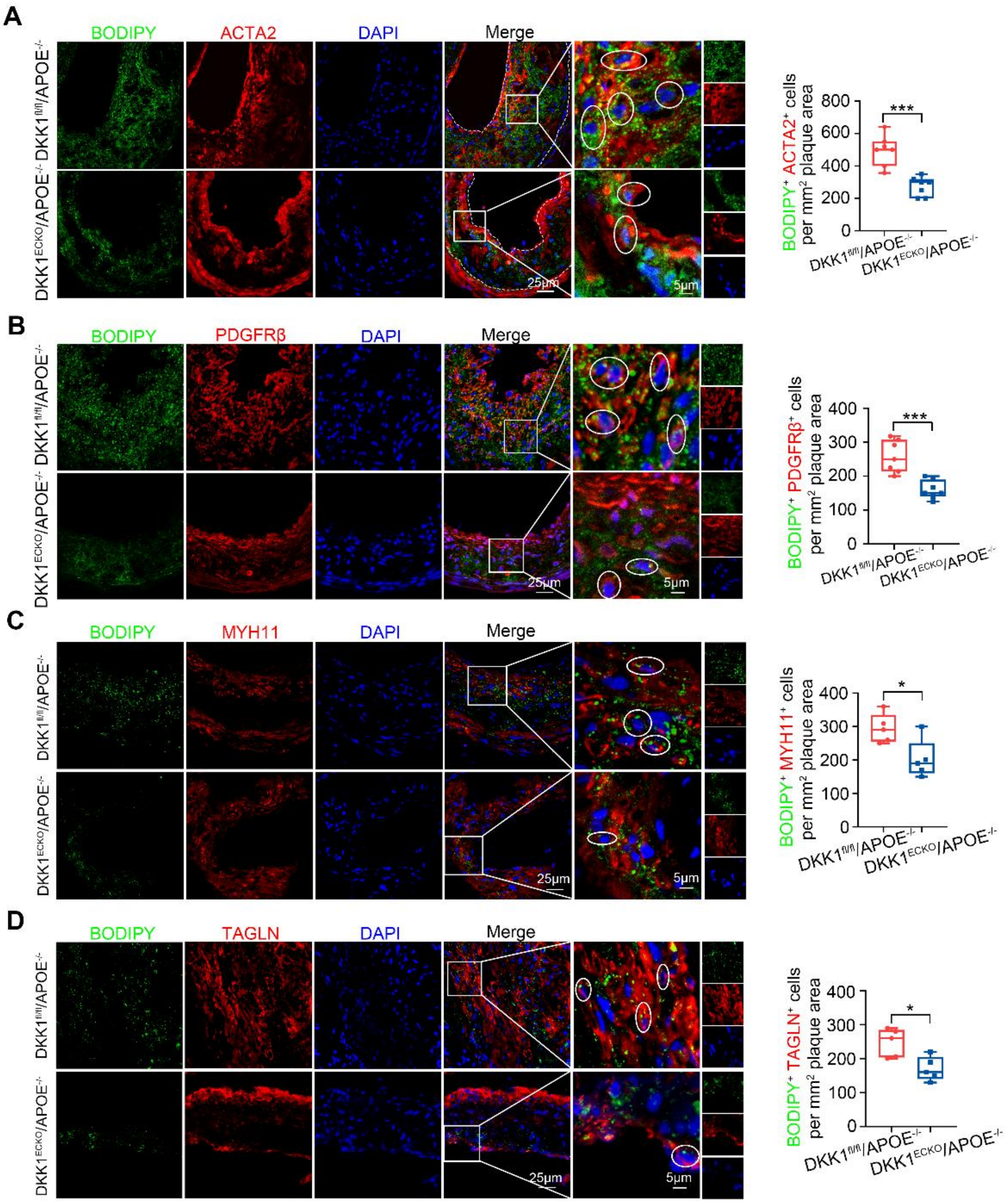

Figure S6 Endothelial specific knockout of DKK1 inhibited SMC-derived foam cell formation in atherosclerosis plaque. (A, B) Confocal fluorescence images of ACTA2 (A, red) or PDGFRβ (B, red) and BODIPY493/503 (green particles) in carotid plaque sections obtained from DKK1$^{fl/fl}$/APOE$^{-/-}$ and DKK1^{ECKO}/APOE$^{-/-}$ mice. White circles indicate SMC-derived foam cells. The number of ACTA2$^+$/BODIPY493/503$^+$ cells and PDGFRβ$^+$/BODIPY493/503$^+$ cells of per mm^2 plaque in two groups was quantified (n = 7). **(C, D)** Representative confocal fluorescence images of MYH11 (C, red) or TAGLN (D, red) and BODIPY493/503 (green particles) in carotid plaque sections obtained from DKK1$^{fl/fl}$/APOE$^{-/-}$ and

$DKK1^{ECKO}/APOE^{-/-}$ mice. White circles indicate SMC-derived foam cells. The number of $MYH11^{+}$/BODIPY493/503^{+} cells or $TAGLN^{+}$/BODIPY493/503^{+} cells of per mm^2 plaque area in two groups was quantified (n = 5). $^{*}p < 0.05$, $^{***}p < 0.001$. Data are shown as the Mean ± SD. All data were analyzed using two-tailed unpaired Student's *t*-test.

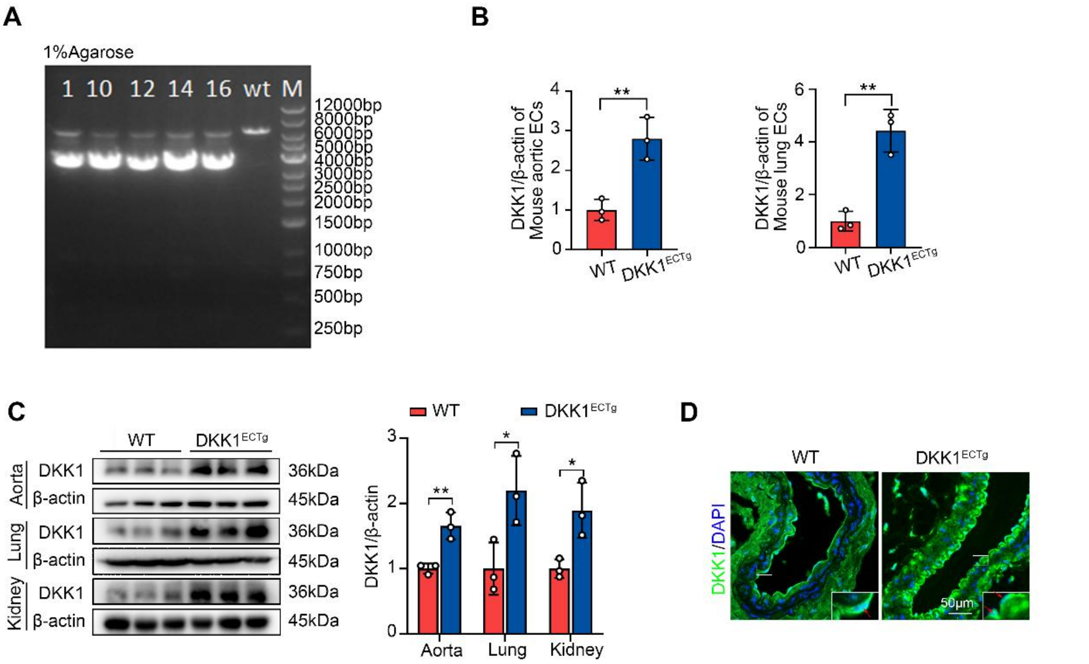

Figure S7 Identification of Endothelial Cells-specific DKK1 transgenic mice (DKK1^{ECTg}). (A) Representative 1% agarose gel image of DKK1^{ECTg} and WT mice. **(B)** Quantitative analysis of DKK1 in primary mouse aortic ECs and primary mouse lung ECs from WT and DKK1^{ECTg} mice (n = 3). **(C)** Representative Western blot images and analysis for DKK1 in aorta, lung and kidney tissues from WT and DKK1^{ECTg} mice (n = 3). **(D)** Carotid arteries from WT or DKK1^{ECTg} mice were isolated for immunofluorescence staining against DKK1 (green). $^{*}p < 0.05$, $^{**}p < 0.01$. Data are shown as the Mean ± SD. *p* values were calculated using two-tailed unpaired Student's *t*-test.

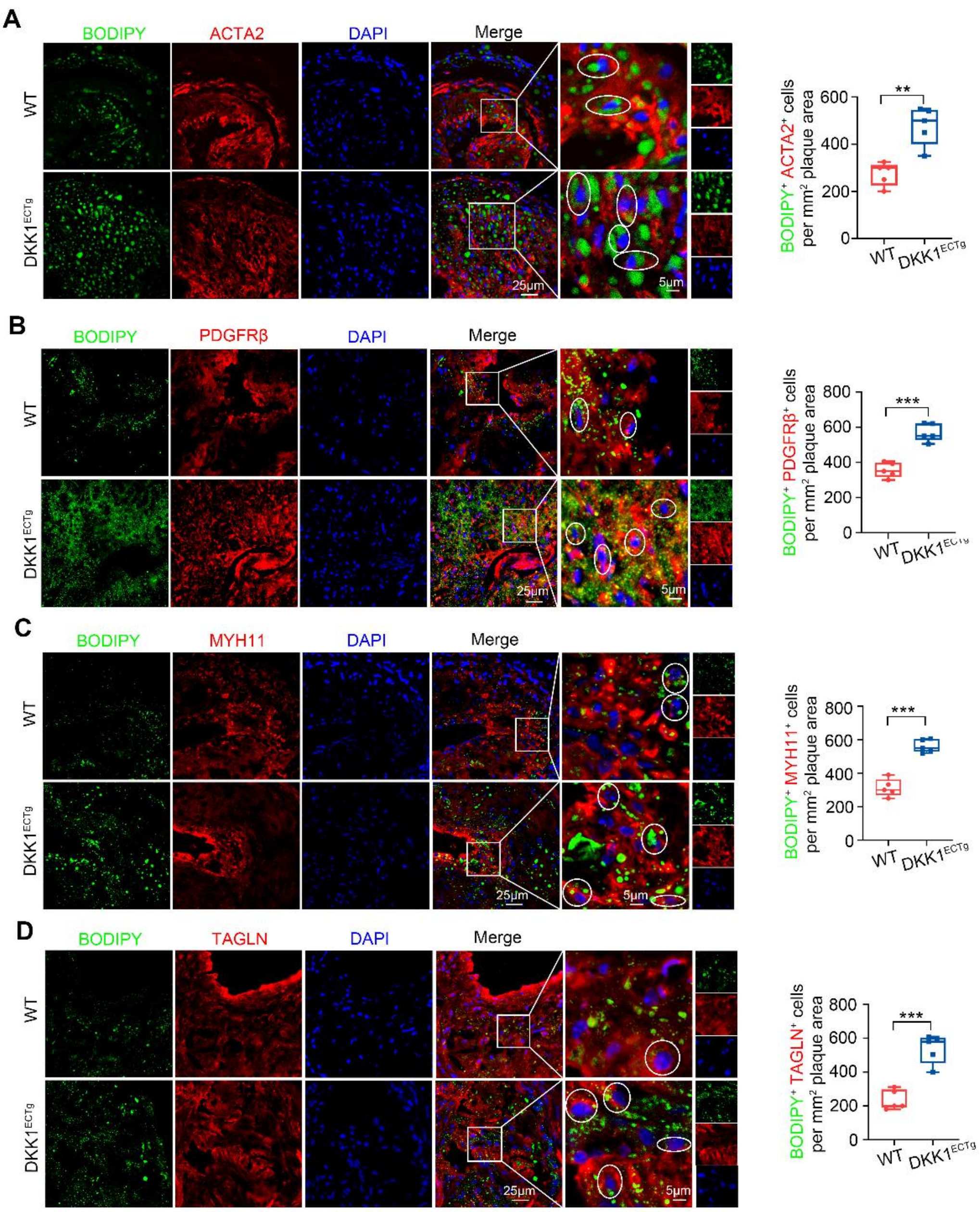

Figure S8 DKK1 overexpression in endothelial promoted SMC-derived foam cell formation in atherosclerosis plaque. (A, B) Confocal double fluorescent staining for ACTA2 (A, red) or PDGFRβ (B, red) and BODIPY493/503 (green particles) in carotid plaque from WT and DKK1^{ECTg} mice to indicate SMC-derived foam cells (white circles). The number of ACTA2^{+}/BODIPY493/503^{+} cells or PDGFRβ^{+}/ BODIPY493/503^{+} cells of per mm^{2} plaque area in two groups was quantified and analyzed (n = 5). **(C, D)** Confocal double fluorescent staining for MYH11 (C, red) or TAGLN (D, red) and BODIPY493/503 (green particles) in carotid plaque from WT

and DKK1^{ECTg} mice to indicate SMC-derived foam cells (white circles). The number of MYH11$^+$/BODIPY493/503$^+$ cells or TAGLN$^+$/BODIPY493/503$^+$ cells of per mm^2 plaque area in two groups was quantified and analyzed (n = 5). $^{**}p < 0.01$, $^{***}p < 0.001$. Data are shown as the Mean ± SD. Statistical analysis was performed using two-tailed unpaired Student's *t*-test.

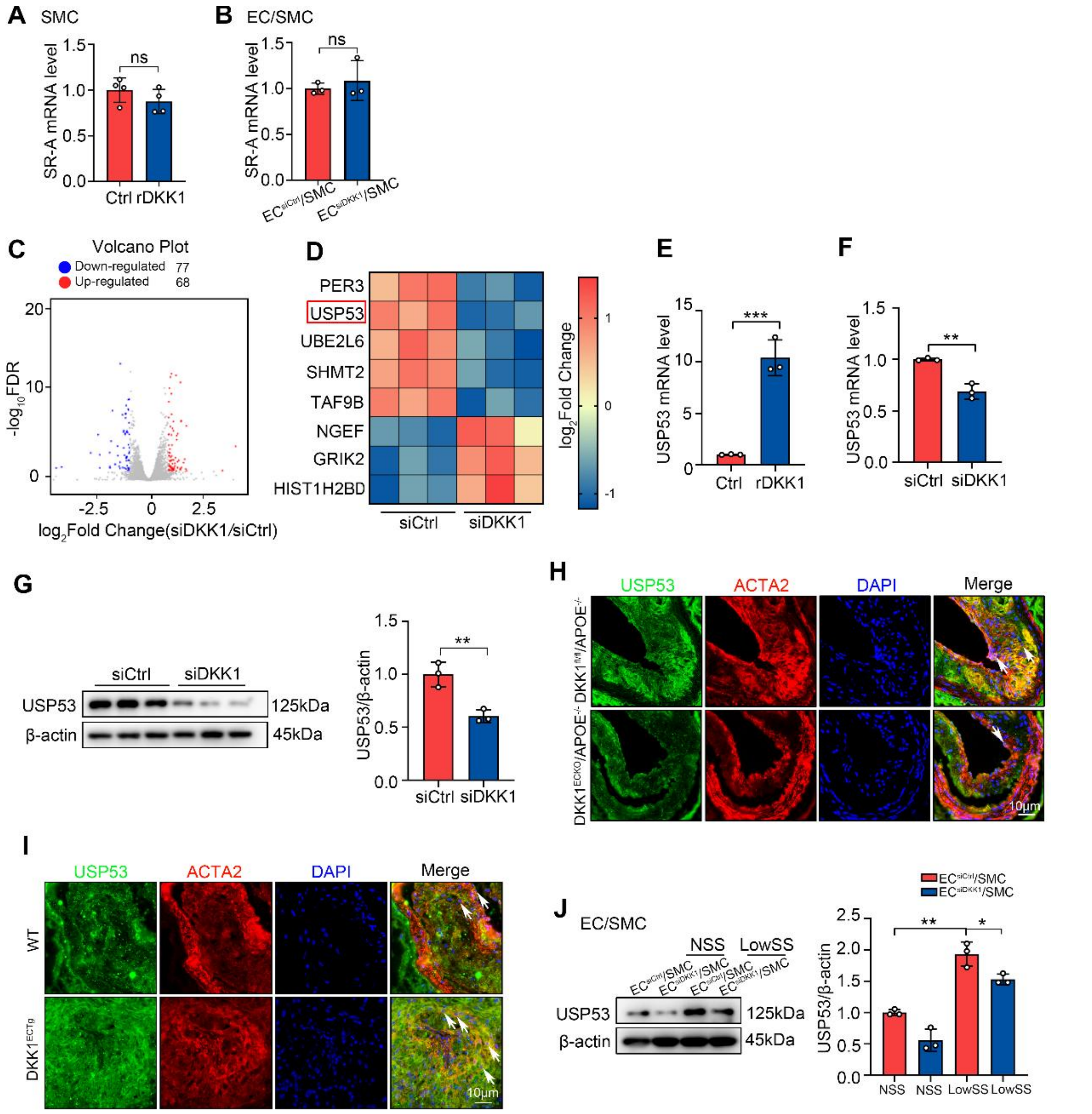

Figure S9 DKK1 regulated the expression of Ubiquitin-specific Protease 53 (USP53). (A) mRNA level of SR-A in HASMCs with rDKK1 incubation (n = 4). **(B)** mRNA level of SR-A in HASMCs co-cultured with ECs that were transfected with siCtrl or siDKK1 under LowSS condition (n = 3). **(C, D)** HASMCs transfected with siCtrl or siDKK1 were analyzed RNA sequencing. Screening result of differentially expressed genes (DEGs) was shown in Volcano Plot (C). Among them, selected genes involved in ubiquitin-proteasome system were listed as a heatmap (D). **(E, F)** mRNA levels of USP53 in HASMCs with or without rDKK1 incubation (E) and transfected with siCtrl or siDKK1 (F) (n = 3). **(G)** Protein expression and statistical analysis of USP53 in HASMCs transfected with siCtrl or siDKK1 (n = 3). **(H, I)** Double

immunofluorescence staining for USP53 (green) and ACTA2 (red) in carotid plaque from DKK1$^{fl/fl}$/APOE$^{-/-}$ and DKK1^{ECKO}/APOE$^{-/-}$ mice or WT and DKK1^{ECTg} mice. **(J)** Representative Western blot image and quantitative analysis for USP53 in HASMCs co-cultured with HAECs that transfected with siCtrl or siDKK1 under shear stress (n = 3). $^{*}p < 0.05$, $^{**}p < 0.01$, $^{***}p < 0.001$, ns not significant. Data are shown as the Mean ± SD. Statistical analysis was performed with Two-tailed unpaired Student's *t*-test or One-way ANOVA followed by Tukey's multiple comparison test.

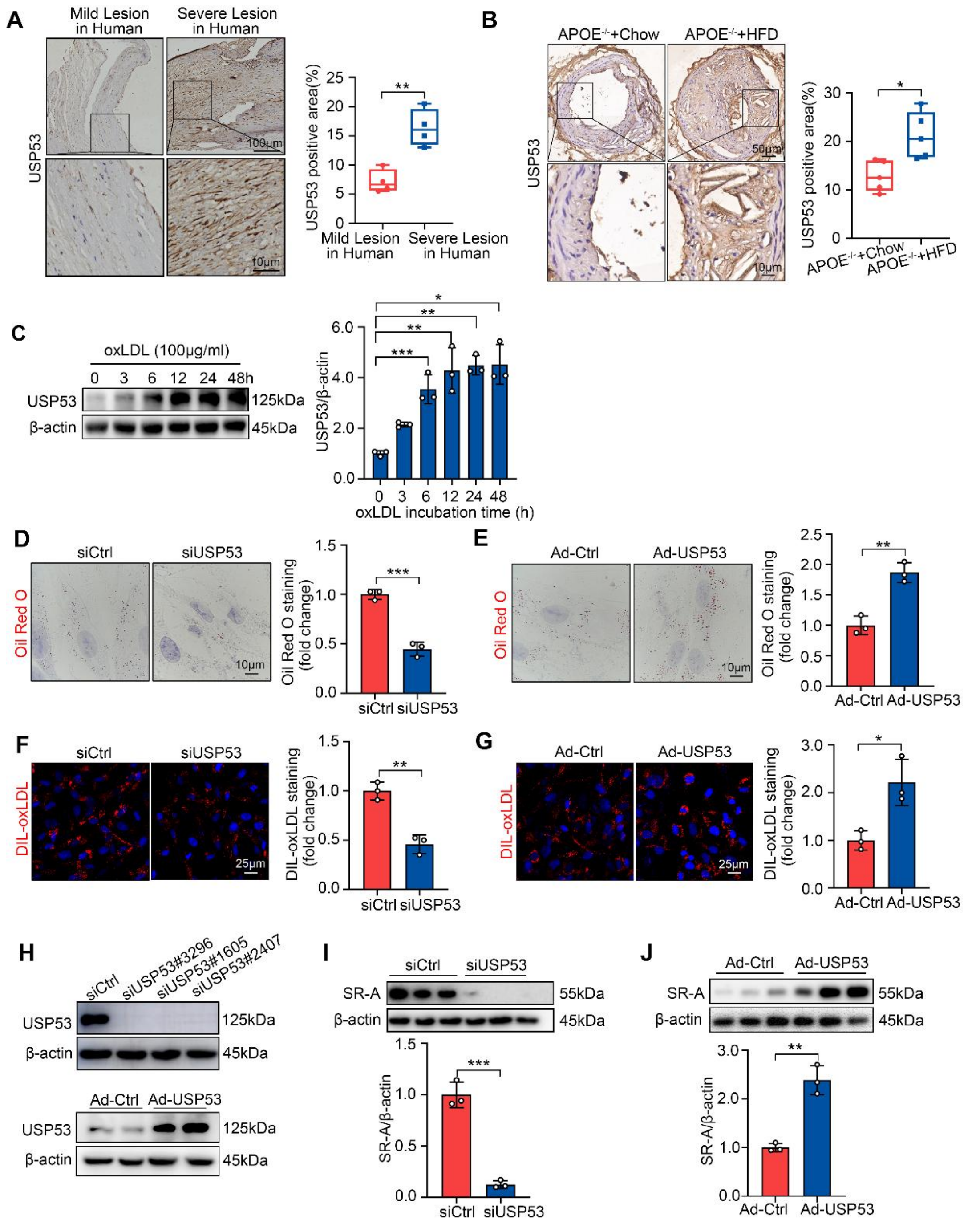

Figure S10 USP53 was involved in atherosclerotic plaque, promoting SR-A expression and foam cell formation in HASMCs. (A) Immunohistochemical staining for USP53 in mild and severe human carotid plaque (n = 4). **(B)** Immunohistochemical detection for USP53 in APOE$^{-/-}$ mice fed with normal Chow Diet or HFD for 8 weeks (n = 5). **(C)** Protein expression and corresponding statistical analysis of USP53 was assessed by Western blot in HASMCs incubated with oxLDL (100 μg/ml) for the indicated time points (n = 3). **(D, E)** Representative optical images

and analysis of Oil Red O staining in HASMCs transfected with siCtrl or siUSP53 (D, n = 3) an Ad-Ctrl or Ad-USP53 (E, n = 3). **(F, G)** Total uptake of DIL-oxLDL in HASMCs with the indicated groups as above (n = 3). **(H)** Western blot images of interference efficiency of USP53 by siRNAs in HASMCs (upper panel). Overexpression of USP53 in HASMCs using adenovirus and proteins were analyzed by Western blot (lower panel). **(I)** Representative Western blot images and quantitative analysis for SR-A in HASMCs transfected with siCtrl or siUSP53 (n = 3). **(J)** Representative Western blot images and quantitative analysis for SR-A in HASMCs transfected with Ad-Ctrl or Ad-USP53 (n = 3). $^{*}p < 0.05$, $^{**}p < 0.01$, $^{***}p < 0.001$. Data are shown as the Mean ± SD. Statistical analysis was performed with Two-tailed unpaired Student's *t*-test or One-way ANOVA followed by Tukey's multiple comparison test.

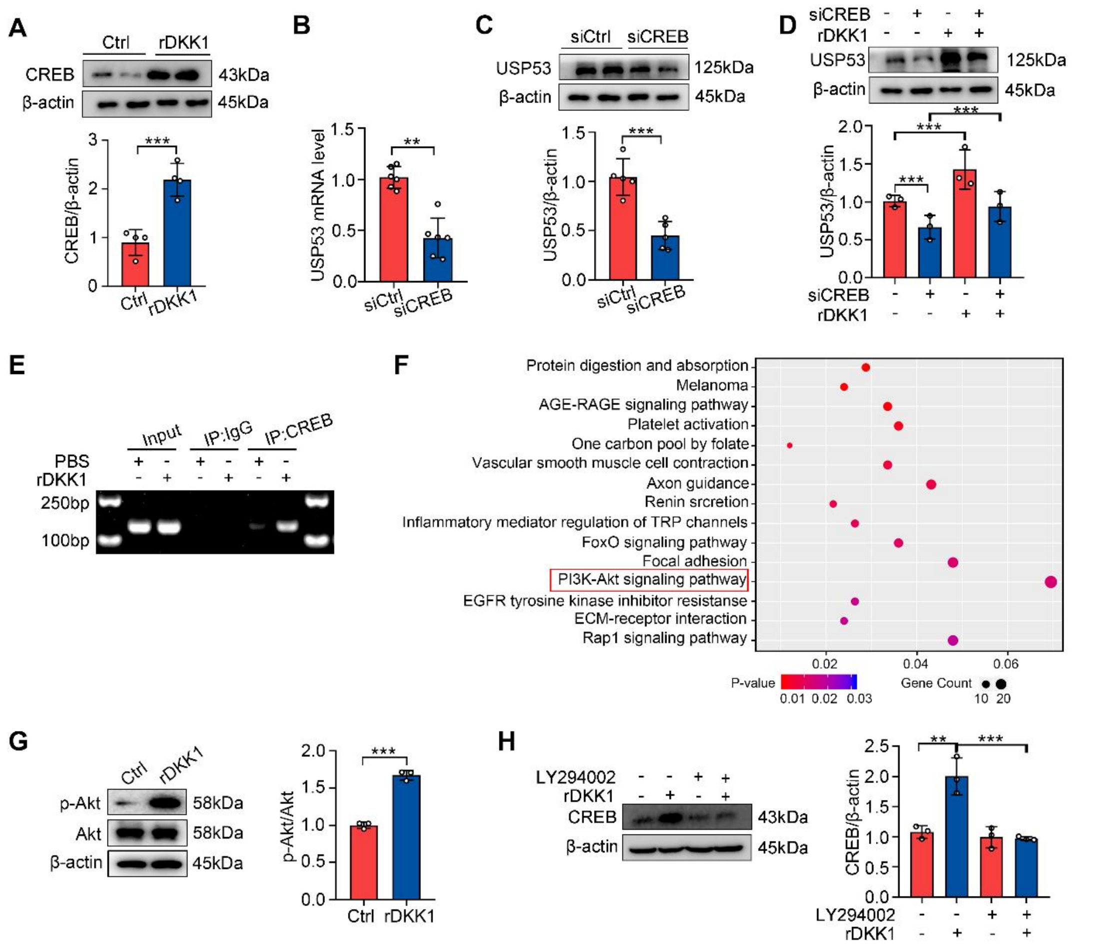

Figure S11 DKK1 regulated USP53 transcription through PI3K/AKT-mediated binding of CREB to the USP53 promoter. (A) Western blot of CREB protein expression in HASMCs treated with rDKK1 (100 ng/mL) or PBS for 24 h (n = 4). **(B, C)** Quantitative RT-qPCR and Western blot analysis of USP53 mRNA (n = 6) and protein (n = 5) expression in HASMCs with knockdown of CREB. **(D)** Western blot analysis of USP53 protein expression in HASMCs with rDKK1 stimulation and CREB knockdown (n = 3). **(E)** ChIP assay to verify the binding of CREB to the USP53 promoter with or without rDKK1 stimulation. **(F)** Top 15 enriched pathways based on the Kyoto Encyclopedia of Genes and Genomes (KEGG) database. **(G)** Phosphorylation levels of Akt in HASMCs after incubation with or without rDKK1 for 24 h (n = 3). **(H)** Western blot analysis of CREB protein expression in HASMCs treated with vehicle control, AKT inhibitor (LY294002), rDKK1 or a combination of the two (n = 3). $^{**}p < 0.01$, $^{***}p < 0.001$. Data are shown as the Mean ± SD. Statistical

analysis was performed with Two-tailed unpaired Student's *t*-test or One-way ANOVA followed by Tukey's multiple comparison test.

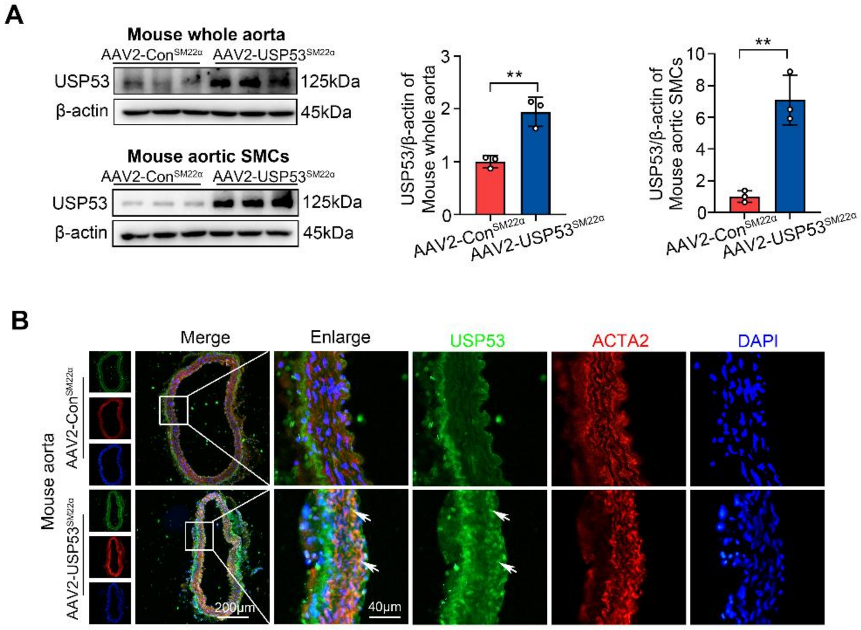

Figure S12 Evaluation of USP53 overexpression in SMCs from AAV2-Con$^{SM22\alpha}$ and AAV2-USP53$^{SM22\alpha}$ mice. (A) Western blot for USP53 in mouse whole aorta tissues and primary mouse aortic SMCs from AAV2-Con$^{SM22\alpha}$ and AAV2-USP53$^{SM22\alpha}$ mice (n = 3 each). **(B)** Double immunofluorescence staining for USP53 (green) and ACTA2 (red) in mouse aorta from AAV2-Con$^{SM22\alpha}$ and AAV2-USP53$^{SM22\alpha}$ mice. White arrows indicate the overlap of USP53 and ACTA2 staining. $^{**}p < 0.01$. Data are shown as the Mean ± SD. *p* values were calculated by two-tailed unpaired Student's *t*-test.

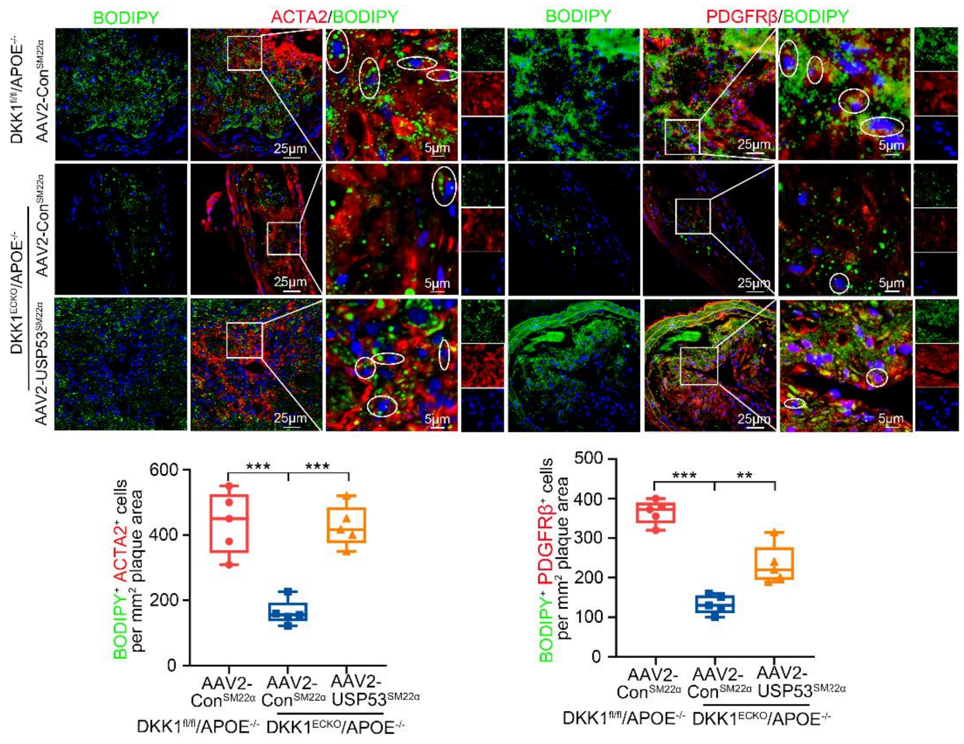

Figure S13 SMCs-specific overexpression of USP53 reversed the reduction of SMC-derived foam cells in DKK1^{ECKO}/APOE$^{-/-}$ mice. Representative confocal fluorescent staining for ACTA2 (red) or PDGFRβ (red) and BODIPY493/503 (green particles) of carotid plaque from three groups of mice (DKK1$^{fl/fl}$/APOE$^{-/-}$+AAV2-Con$^{SM22\alpha}$ group, DKK1^{ECKO}/APOE$^{-/-}$+AAV2-Con$^{SM22\alpha}$ group, DKK1^{ECKO}/APOE$^{-/-}$+AAV2-USP53$^{SM22\alpha}$ group) (n=5 per group). $^{**}p < 0.01$, $^{***}p < 0.001$. Data are shown as the Mean ± SD. p values were calculated by two-tailed unpaired Student's t-test.

Supplementary Tables

Table S1. Metabolic profile in DKK1$^{fl/fl}$/APOE$^{-/-}$ and DKK1^{ECKO}/APOE$^{-/-}$ mice

Characteristics	DKK1$^{fl/fl}$/APOE (n=7)	DKK1^{ECKO}/APOE$^{-/-}$ (n=7)	*p* value
BW (g)	30.800 ± 1.083	30.229 ± 0.429	0.6579
TC (mmol/L)	23.799 ± 0.963	21.970 ± 0.828	0.2543
TG (mmol/L)	0.974 ± 0.034	1.193 ± 0.082	0.0523
HDL-c (mmol/L)	1.710 ± 0.169	1.856 ± 0.248	0.6936
LDL-c (mmol/L)	7.787± 0.346	7.146 ± 0.310	0.0555

BW, body weight; TC, total cholesterol; TG, triglycerides; HDL-c, high-density lipoprotein cholesterol; LDL-c, low-density lipoprotein cholesterol. n=7 per group. Two-tailed unpaired Student's *t*-test was used for statistical analysis. There was no statistically significant difference in these characteristics between the two groups of mice. Data were presented as Mean ± SEM.

Table S2. Metabolic profile in WT and DKK1^{ECTg} mice

Characteristics	WT (n=7)	DKK1^{ECTg} (n=7)	*p* value
BW (g)	29.290 ± 0.761	28.786 ± 0.722	0.6580
TC (mmol/L)	19.290 ± 0.583	19.999 ± 0.648	0.4661
TG (mmol/L)	0.881 ± 0.078	0.919 ± 0.087	0.7738
HDL-c (mmol/L)	1.469 ± 0.068	1.424 ± 0.071	0.6838
LDL-c (mmol/L)	5.909 ± 0.281	6.210 ± 0.164	0.4079

BW, body weight; TC, total cholesterol; TG, triglycerides; HDL-c, high-density lipoprotein cholesterol; LDL-c, low-density lipoprotein cholesterol. n=7 per group. Two-tailed unpaired Student's *t*-test was used for statistical analysis. There was no statistically significant difference in these characteristics between the two groups

of mice. Data were presented as Mean ± SEM.

Table S3. siRNA sequences used in this study

Gene	siRNA sequence (sense/antisense)
DKK1 siRNA (homo)	sense: GCUUCACACUUGUCAGAGATT<br>antisense: UCUCUGACAAGUGUGAAGCCT
CKAP4 siRNA (homo-1923)	sense: GGAAUGAUCUGGAUAGGUUTT<br>antisense: AACCUAUCCAGAUCAUUCCTT
LRP6 siRNA (homo-305)	sense: GCGGUGGACUUUGUGUUUATT<br>antisense: UAAACACAAAGUCCACCGCTT
LRP6 siRNA (homo-2060)	sense: GCAGAUAUCAGACGAAUUUTT<br>antisense: AAAUUCGUCUGAUAUCUGCTT
LRP6 siRNA (homo-4793)	sense: GCAACAGCCAAGGGCUAUATT<br>antisense: UAUAGCCCUUGGCUGUUGCTT
USP53 siRNA (homo-3296)	sense: GAGCCAACAUCACUUAGAATT<br>antisense: UUCUAAGUGAUGUUGGCUCTT
USP53 siRNA (homo-1605)	sense: GUGCGGUACAUUUCUACAATT<br>antisense: UUGUAGAAAUGUACCGCACTT
USP53 siRNA (homo-2407)	sense: GUGCUCUGAAAGCUAUUGATT<br>antisense: UCAAUAGCUUUCAGAGCACTT
SR-A siRNA (homo-1071)	sense: GGGAGUGGAAACACAUUAATT<br>antisense: UUAAUGUGUUUCCACUCCCTT
SR-A siRNA (homo-704)	sense: GGAGCGUGUUUACAAUGUATT<br>antisense: UACAUUGUAAACACGCUCCTT
SR-A siRNA (homo-841)	sense: CUCAGACCUUGAGAAAUAUTT<br>antisense: AUAUUUCUCAAGGUCUGAGTT
CREB siRNA (homo)	sense: GAGAGAGGUCCGUCUAAUGTT<br>antisense: CAUUAGACGGACCUCUCUCTT

Table S4. The primer sequences used in this study

Gene	Sequence (5'-3')
Human DKK1	Forward: CGGGCGGGAATAAGTACCAG<br>Reverse: GGGACTAGCGCAGTACTCATC
Human SR-A	Forward: CACGAGGATTTCCAGGTCCAA<br>Reverse: ATCAGTGAGTTGTACTGGTCTTAAT
Human USP53	Forward: GCTCCTCTCATCCAGCAACAA<br>Reverse: GGGGTGGTAGATTCTGGCAAA

Table S5. Antibodies of Western blot used in this study

Antibody	Source	Cat NO.
DKK1	SantaCruz	sc-374574
DKK1	Sigma-Aldrich	SAB1404944
DKK1	Proteintech Group	21112-1-AP
CD36	abcam	ab133625
LOX-1	abcam	ab214427
SR-A	abcam	ab151707
SR-A	abcam	ab271070
β-actin	Proteintech Group	66009-1-Ig
CKAP4	abcam	ab152154
LRP6	Cell Signaling Technology	3395T
USP53	Abclonal	A14353
USP53	Origene	TA808887
CREB	Cell Signaling Technology	9197S
Myc	Cell Signaling Technology	2276S
Flag	Cell Signaling Technology	8146S
Flag	Cell Signaling Technology	14793S
HA	Origene	TA180128
HA	Cell Signaling Technology	5017S
Ubquitin	Cell Signaling Technology	3936
Ubquitin	Cell Signaling Technology	3933
K63-linkage specific Polyubiquitin	Cell Signaling Technology	12930S
K48- linkage specific Polyubiquitin	Cell Signaling Technology	12805S
Akt	abcam	ab182729
p-Akt	Cell Signaling Technology	4060T

Normal Rabbit IgG	abcam	ab172730
